# Promiscuous terpene synthases from *Prunella vulgaris* highlight the importance of substrate and compartment switching in terpene synthase evolution

**DOI:** 10.1111/nph.15778

**Published:** 2019-04-08

**Authors:** Sean R. Johnson, Wajid Waheed Bhat, Radin Sadre, Garret P. Miller, Alekzander Sky Garcia, Björn Hamberger

**Affiliations:** ^1^ Department of Biochemistry and Molecular Biology Michigan State University East Lansing MI 48824 USA; ^2^ Department of Pharmacology and Toxicology Michigan State University East Lansing MI 48824 USA

**Keywords:** diterpenoid, *Prunella vulgaris* (common selfheal), terpene synthase, transcriptome, transit peptide, vulgarisane

## Abstract

- The mint family (Lamiaceae) is well documented as a rich source of terpene natural products. More than 200 diterpene skeletons have been reported from mints, but biosynthetic pathways are known for just a few of these.
- We crossreferenced chemotaxonomic data with publicly available transcriptomes to select common selfheal (*Prunella vulgaris*) and its highly unusual vulgarisin diterpenoids as a case study for exploring the origins of diterpene skeletal diversity in Lamiaceae. Four terpene synthases (TPS) from the TPS‐a subfamily, including two localised to the plastid, were cloned and functionally characterised. Previous examples of TPS‐a enzymes from Lamiaceae were cytosolic and reported to act on the 15‐carbon farnesyl diphosphate. Plastidial TPS‐a enzymes using the 20‐carbon geranylgeranyl diphosphate are known from other plant families, having apparently arisen independently in each family.
- All four new enzymes were found to be active on multiple prenyl‐diphosphate substrates with different chain lengths and stereochemistries. One of the new enzymes catalysed the cyclisation of geranylgeranyl diphosphate into 11‐hydroxy vulgarisane, the likely biosynthetic precursor of the vulgarisins.
- We uncovered the pathway to a rare diterpene skeleton. Our results support an emerging paradigm of substrate and compartment switching as important aspects of TPS evolution and diversification.

## Introduction

Diterpenoids are an important and diverse class of specialised metabolites in plants, particularly abundant in the mint family (Lamiaceae). The committed step in diterpenoid biosynthesis is the cyclisation of a 20‐carbon poly‐isoprene diphosphate, usually (*E*,*E*,*E*)‐geranylgeranyl diphosphate (GGPP), by one or two terpene synthase enzymes (TPSs). The resulting diterpenoid core ring structure, or skeleton, can then be modified by a series of decorating enzymes, such as cytochromes P450 and acyl‐transferases. We recently reported (Johnson *et al*., 2019), that just a few taxonomically widespread diterpene skeletons, including the kaurane, labdane, abietane, and clerodane, account for most of the known diterpene structures in Lamiaceae. All the diterpene synthases (diTPSs) characterised so far from Lamiaceae are involved in the biosynthesis of these widespread skeleton types. However, within Lamiaceae, biosynthetic pathways to over 200 less widespread diterpene skeletons remain unknown. Many of these rarer skeletons seem to be confined to a single genus or species (Johnson *et al*., 2019). While some of the skeletons are likely to arise from reactions occurring after the TPS‐mediated step, others may come from as‐yet undiscovered diTPS activities. Finding examples of TPS responsible for rare cyclisations would help to clarify the process of TPS functional divergence and the evolutionary basis for diterpene skeletal diversity.

**Figure 1:**
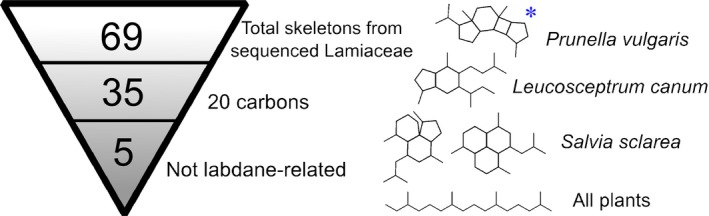
Summary of diterpene skeletons from mints with available transcriptomes. The five nonlabdane‐related skeletons are shown on the right. The vulgarisane skeleton is indicated by the blue asterisk.

To identify diTPS genes encoding enzymes responsible for unusual cyclisations, we crossreferenced data derived from the Dictionary of Natural Products v.26.2, and the NCBI Sequence Read Archive (SRA) to generate a list of diterpene skeletons found in Lamiaceae species with publicly available transcriptome data. Of the 69 total skeletons from species from which transcriptome data were available (Supporting Information Dataset S1), 34 were not C_20_, indicating that they are formed by skeletal modifications downstream of the diTPS‐catalysed reactions. All but five of the C_20_ skeletons appeared to be labdane‐related, featuring a decalin core or an obvious derivative. As biosynthetic pathways for labdane‐related diterpenoids in Lamiaceae have already received considerable attention, we decided to focus on a more unusual skeleton. Of the five options (Laville *et al*., 2012; Luo *et al*., 2012; Lou *et al*., 2014), we chose to investigate the biosynthesis of the vulgarisane skeleton (Fig. 1) from *Prunella vulgaris* because live plants were accessible and there was high‐quality transcriptome data available for both root and leaf tissue (Xiao *et al*., 2013; Boachon *et al*., 2018). *Prunella vulgaris* has a long history of use in traditional Chinese and Unani medicine (Rasool *et al*., 2010; Bai *et al*., 2016), with reported antimicrobial, antiviral, and other therapeutic applications.

**Figure 2:**
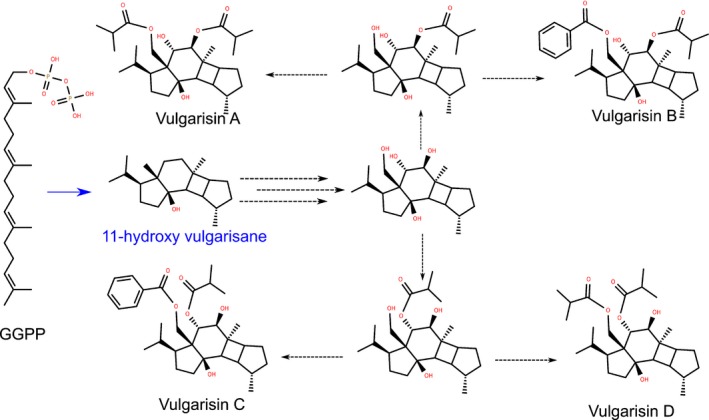
Proposed pathway from geranylgeranyl diphosphate (GGPP) to vulgarisanes A–D. Blue indicates the step sought in this study, which was found to be catalysed by PvHVS.

The vulgarisane skeleton has only been reported from *P. vulgaris* (Lou *et al*., 2014, 2017), in the form of vulgarisins A–D, and from the marine sponge *Hippospongia lachne* (Hong *et al*., 2017). This class of these compounds was reported to have potential antiallergic (Hong *et al*., 2017), antigout (Chinese patent CN105456245A), or blood pressure‐lowering properties (Chinese patent CN106562948A). The lack of a decalin core in the vulgarisane skeleton suggested that the enzyme catalysing the cyclisation step may be a diTPS outside the canonical TPS‐c and TPS‐e subfamilies that include all known Lamiaceae diTPSs (Johnson *et al*., 2019). Among TPS candidates from the roots, were a group of proteins from the TPS‐a subfamily. In Lamiaceae, all characterised enzymes from the TPS‐a subfamily are cytosolic sesquiterpene synthases (sesquiTPSs), which use a C_15_‐prenyl‐diphosphate substrate. However, there are a few examples of TPS‐a diTPSs harbouring putative plastid transit peptides from Euphorbiaceae (Mau & West, 1994; Kirby *et al*., 2010; King *et al*., 2014; Luo *et al*., 2016), Solanaceae (Ennajdaoui *et al*., 2010), and Brassicaceae (Vaughan *et al*., 2013; Wang *et al*., 2016) that catalyse the cyclisation of GGPP into nonlabdane‐related diterpenes. Certain individual TPS‐a candidates from *P. vulgaris* contained putative plastidial transit peptides, while others were predicted to be cytosolic. We cloned open reading frames encoding two plastidial and two cytosolic TPS candidates. Multiple strategies were used to characterise the activities of the encoded enzymes, including transient expression in *Nicotiana benthamiana* and *in vitro* assays using purified recombinant enzyme. Enzyme activity was tested against six distinct prenyl‐diphosphates: GGPP, nerylneryl diphosphate (NNPP, (*Z*,*Z*,*Z*)‐GGPP), (*E*,*E*)‐farnesyl diphosphate ((*E*,*E*)‐FPP), (Z,Z)‐farnesyl diphosphate ((*Z*,*Z*)‐FPP), (*E*)‐geranyl diphosphate (GPP), and neryl diphosphate (NPP, (*Z*)‐GPP). All four enzymes showed activity on substrates of multiple chain lengths. Notably, one of the enzymes, PvHVS, converted GGPP to the diterpene 11‐hydroxy vulgarisane, previously proposed (Lou *et al*., 2014) as the precursor to vulgarisins A–D (Fig. 2). Phylogenetic analysis of the new clones from *P. vulgaris* together with TPS‐a enzymes from other plants suggests that the acquisition of a plastid transit peptide and diTPS activity occurred independently at least four times in dicot lineages, consistent with an overall pattern of compartment and substrate switching playing important roles throughout the evolution of TPSs.

## Materials and Methods

### Plant materials

Leaf and root samples were collected from *P. vulgaris* plants obtained from the W. J. Beal Botanical Garden at Michigan State University.

### Generating a list of Lamiaceae transcriptomes and skeletons

In a recent study, we described the extraction of diterpene skeletons from Lamiaceae diterpene structures obtained from the Dictionary of Natural Products (http://dnp.chemnetbase.com). Critically, we had associated each skeleton to TaxIDs from the NCBI taxonomy database (Federhen, 2012) of the species where the skeleton occurs (Johnson *et al*., 2019). Here we extend the previous work by crossreferencing our list of Lamiaceae diterpene skeletons to a list of Lamiaceae species with transcriptome data available in the NCBI SRA. Each record in the SRA is already associated with a TaxID, so the crossreferencing was trivial.

### *Prunella vulgaris* transcriptome analysis

*P. vulgaris* root transcriptome reads and assembly from Illumina HiSeq 2000 (SRA: SRR766790) were downloaded from the NCBI SRA (https://www.ncbi.nlm.nih.gov/sra) and the PhytoMetSyn project (Xiao *et al*., 2013) (https://bioinformatics.tugraz.at/phytometasyn/). Reads were trimmed based on quality and presence of adapter fragments using Bbduk (v.37.9, Bushnell, https://sourceforge.net/projects/bbmap/), and expression levels were calculated using salmon (v.0.11.2) (Patro *et al*., 2017). Peptide sequences were extracted using transdecoder (v.4.1.0) (Haas *et al*., 2013). A leaf transcriptome assembly, derived from Illumina HiSeq 2500 reads, was obtained from the Mint Evolutionary Genomics Consortium (SRA: SRR5150718) (Boachon *et al*., 2018). Candidate TPSs from both tissues were identified using blastp (v.2.2.31+) searches against a set of reference TPSs, hits with < 60% coverage of the reference sequence were discarded. targetp (v.1.1b) (Emanuelsson *et al*., 2000) was used to predict the localisation of all candidate genes.

The publicly available root assembly appeared to have fragmented contigs of some of the TPS genes, including the one we cloned as *PvTPS4*. We tried to generate an improved assembly by incorporating long reads from an Oxford Nanopore GridIon instrument (Oxford, UK) (Method S1).

### Phylogenetic tree

Transit peptides were predicted by targetp (v.1.1) and removed before alignment. Peptide sequences were aligned using clustal omega (v.1.2.1) (Sievers *et al*., 2011) and a maximum likelihood tree was generated using raxml (v.8.2.11) (Stamatakis, 2014) with automatic model selection and 1000 bootstrap iterations. The tree graphic was rendered using ETE3 (Huerta‐Cepas *et al*., 2016). Sequences are given in Dataset S3 (see later).

### Cloning and sources of genes used

All primers used are listed in Table S1. *P. vulgaris* root total RNA was extracted using a previously described method (Hamberger *et al*., 2011). cDNA was synthesised using RevertAid First Strand cDNA Synthesis Kit (Thermo Fisher Scientific, Waltham, MA, USA) with oligo(dT) primers.

All four TPS candidate coding sequences were cloned into the plant expression vector pEAQ‐HT (Sainsbury *et al*., 2009) for transient expression assays in *N. benthamiana*. For consistency and to avoid background activity from endogenous sesquiterpene synthases, *PvTPS4* and *PvTPS5*, which lack a putative transit peptide, were cloned into pEAQ‐HT with the addition of an N‐terminal transit peptide derived from *Arabidopsis thaliana* RubisCO small subunit (Sadre *et al*., 2019) (GenBank: NP_176880.1, first 54 amino acids). *PvTPS2* and *PvHVS* were cloned without modification into pEAQ‐HT. *Solanum habrochaites (Z*,*Z)‐FPP synthase* (GenBank: ACJ38408.1) (Sallaud *et al*., 2009) was also cloned into the pEAQ‐HT vector. *Solanum lycopersicum NNPP synthase* (GenBank: JX943884.1) (Akhtar *et al*., 2013) was cloned from leaf cDNA into the pEAQ‐HT vector. Both (*Z*,*Z*)‐FPP synthase and NNPP synthase contain putative plastid transit peptides in their native form.

The open reading frames encoding the four TPS candidates were also cloned into pET28b+ (Novagen, Burlington, MA, USA) for expression in *Escherichia coli*, for either *in vivo* assays when co‐expressed with other pathway genes, or for purification of the recombinant protein for *in vitro* assays. *PvTPS2* and *PvHVS* were cloned into pET28b+ as N‐terminal truncations to remove the predicted plastid transit peptide, 48 and 43 amino acids, respectively. *NNPP synthase* (Akhtar *et al*., 2013) was also cloned as an N‐terminal truncation, removing 51 amino acids, into an *E. coli* expression vector. *NNPP synthase* was cloned into the pACYCDuet vector by cutting *GGPP synthase* out of pGG (Cyr *et al*., 2007) through digestion with *Nde*I and *Xho*I, and inserting the truncated *NNPP synthase* in its place using InFusion cloning (TaKaRa Bio, Mountain View, CA, USA). We named this vector pNN.

### *In vitro* assays

pET28b+ plasmids containing N‐terminal truncated *PvHVS* or *PvTPS2*, or full‐length *PvTPS4* or *PvTPS5* were transformed separately into *E. coli* C41 OverExpress cells. Primary cultures of each transformant were grown overnight in 5 ml LB with 50 μg ml^−1^ kanamycin, and 500 μl of this culture was subsequently added to inoculate 50 ml LB with 50 μg ml^−1^ kanamycin. Cultures were grown at 37°C and 180 rpm shaking until an OD_600_ of 0.7 was reached, at which point IPTG was added to a concentration of 0.2 mM and expression was carried out overnight at 16°C. Cells were collected by centrifugation and resuspended in Binding Buffer (20 mM HEPES, pH 7.2, 25 mM imidazole, 500 mM NaCl, and 5% (v/v) glycerol) with 10 μl ml^−1^ protease inhibitor cocktail (Sigma) and 0.1 mg ml^−1^ lysozyme (VWR). Cells were lysed by sonication and lysates were centrifuged at 11 000 ***g*** for 20 min. Supernatants were added to Ni‐NTA columns (GE Healthcare His Spintrap, Chicago, IL, USA), washed with Binding Buffer, eluted twice with Elution Buffer (Binding Buffer with 350 mM imidazole), and desalted on PD MidiTrap G‐25 columns (GE Healthcare) with Desalting Buffer (20 mM HEPES, pH 7.2, 1 mM MgCl_2_, 350 mM NaCl, 5 mM DTT, and 5% (v/v) glycerol).

A typical *in vitro* TPS assay (final volume 500 μl) contained 5 μg substrate, (GPP, NPP, (*E*,*E*)‐FPP, (*Z*,*Z*)‐FPP, or GGPP; NPP and (*Z*,*Z*)‐FPP from Echelon Biosciences (Salt Lake City, UT, USA), others from Cayman Chemical (Ann Arbor, MI, USA)), 200 μg purified enzyme, 10 mM MgCl_2_, 100 mM KCl, 5 mM DTT, and 10% (v/v) glycerol in 50 mM HEPES, pH 7.2, with 500 μl hexane overlay. Reactions were carried out at 30°C for 4 h, followed by vortexing to extract the products into the organic phase. Layers were separated by centrifugation, and hexane layers were removed for GC/MS analysis.

### *Nicotiana benthamiana* expression

Transient expression assays in *N. benthamiana* used methods that we have described in detail elsewhere (Johnson *et al*., 2019; Sadre *et al*., 2019). Mixtures of *Agrobacterium tumefaciens* harbouring TPS candidates in the pEAQ‐HT vector were co‐infiltrated into *N. benthamiana* leaves together with *A. tumefaciens* harbouring *Plectranthus barbatus* (syn *Coleus forskohlii*) 1‐deoxy‐d‐xylose 5‐phosphate synthase (*DXS*) (GenBank: KP889115.1) (Andersen‐Ranberg *et al*., 2016) in the pEarleyGate (Earley *et al*., 2006) vector, along with *A. tumefaciens* harbouring one of the following prenyl transferases: *P. barbatus GGPP synthase* (GenBank: KP889114.1) (Andersen‐Ranberg *et al*., 2016) in pEarleyGate, *A. thaliana (E*,*E)‐FPP synthase* (GenBank: NM_117823.4) (Keim *et al*., 2012) with the addition of an N‐terminal transit peptide (see above, Cloning and sources of genes used) in pEarleyGate, *S. habrochaites (Z*,*Z)‐FPP synthase* (Sallaud *et al*., 2009) in pEAQ‐HT, *S. lycopersicum NNPP synthase* (Akhtar *et al*., 2013) in pEAQ‐HT. Individual *A. tumefaciens* cultures were grown overnight at 28°C, then pelleted by centrifugation at 3800 ***g*** for 10 min. Pelleted cultures were resuspended in buffer (10 mM MES‐KOH pH 5.7, 10 mM MgCl2, 200 μM acetosyringone), diluted to an OD_600_ of 0.8, and incubated for 30 min at 28°C. Cultures containing different constructs were mixed in equal ratios to make up the appropriate combinations before infiltration.

For small‐scale assays, 100 mg of leaf tissue were harvested 5 d after infiltration and extracted overnight with 1 ml hexane, which was then analysed by GC‐MS. At least two independent replicates were performed for each condition. For large‐scale production (Andersen‐Ranberg *et al*., 2016) of 11‐hydroxy vulgarisane for NMR, 15 whole plants were vacuum infiltrated at 100 mBar for 30–60 s with mixtures of *A. tumefaciens* harbouring plasmids containing *DXS*,* GGPP synthase*, and *PvHVS* constructs. The product was purified as described below.

### *Escherichia coli* expression

*E. coli* OverExpress C41 strain (Lucigen, Middleton, WI, USA) was co‐transformed with pIRS (Morrone *et al*., 2010) and pNN to create an NNPP producing *E. coli* strain. This strain was transformed separately with N‐terminally truncated pET28b(+)‐PvHVS, N‐terminally truncated pET28b(+)‐PvTPS2, pET28b(+)‐PvTPS4, or pET28b(+)‐PvTPS5. Transformed *E. coli* cells were grown on LB‐agar plates containing 25 μg ml^−1^ kanamycin, 20 μg ml^−1^ chloramphenicol, and 25 μg ml^−1^ streptomycin, and were further screened for the presence of all the plasmids using colony PCR. Recombinant cultures were grown in 50 ml Terrific Broth medium (pH 7.0), with appropriate antibiotics, in 250 ml Erlenmeyer flasks. The cultures were first grown at 37°C to mid‐log phase (OD_600_ of 0.6), then the temperature dropped to 16°C for 1 h before induction with 1 mM isopropylthiogalactoside (IPTG) and supplementation with 40 mM pyruvate and 1 mM MgCl_2_. The induced cultures were grown for an additional 72 h before extraction with an equal volume of hexane, with the organic phase then separated, concentrated under N_2_ and analysed by GC‐MS.

### GC‐MS

All GC‐MS analyses were performed on an Agilent 7890A GC (Santa Clara, CA, USA) with an Agilent VF‐5 ms column (30 m × 250 μm × 0.25 μm, with 10 m EZ‐Guard) and an Agilent 5975C detector. The inlet was set to 250°C splitless injection of 1 μl, He carrier gas with column flow of 1 ml min^−1^. The detector was activated after a 3‐min solvent delay. For assays with C_15_ and C_20_ substrates, the oven temperature ramp was start at 80°C hold 1 min, increase 40°C min^−1^ to 130°C, increase 10°C min^−1^ to 250°C, increase 100°C min^−1^ to 325°C hold 3 min. For assays with C_10_ substrates, the oven temperature ramp was start at 40°C, increase 10°C min^−1^ to 180°C, increase 40°C min^−1^ to 250°C hold 3 min.

For GC‐MS‐based root metabolomics, 350 mg of fresh roots were cut into 3 mm sections and extracted for 3 h in 1 ml methyl‐tertbutyl ether. The extract was analysed by GC‐MS using the same method as for the enzyme assays.

### Compound purification and NMR

Leaves of *N. benthamiana* (*c*. 78 g, FW) transiently expressing *PvHVS*,* DXS*, and *GGPP synthase* were extracted overnight in 600 ml hexane. The extract was dried down on a rotary evaporator. 11‐hydroxy vulgarisane was purified from the resin using silica gel flash column chromatography (Still *et al*., 1978) with a mobile phase of 5% ethyl‐acetate in hexane. NMR spectra were measured on an Agilent DirectDrive2 500 MHz spectrometer using CDCl_3_ as the solvent. CDCl_3_ peaks were referenced to 7.26 and 77.00 ppm for ^1^H and ^13^C spectra, respectively.

### UHPLC/MS metabolomics

For both root and leaf, 100 mg of fresh tissue and 1 ml 70% methanol were added, mixed and incubated in the dark at room temperature for 16 h. A 10‐μl volume of each extract was subsequently analysed using a 31‐min gradient elution method on an Acquity BEH C18 UHPLC column (2.1 × 100 mm, 1.7 μm; Waters, Milford, MA, USA) with mobile phases consisting of 10 mM ammonium formate (solvent A) and methanol (solvent B). The gradient elution method employed 50% B at 0.00–2 min, linear gradient to 99% B at 30.00 min, followed by a return to 50% B and held from 30.1 to 31 min. The flow rate was 0.3 ml min^−1^ and the column temperature was 40°C. The mass spectrometer (Xevo G2‐XS QTOF; Waters) was equipped with an electrospray ionisation source and operated in positive‐ion mode. Source parameters were as follows: capillary voltage 4.5 kV, cone voltage 40 V, desolvation temperature 300°C, source temperature 100°C, cone gas flow 50 l h^−1^, and desolvation gas flow 600 l h^−1^. Mass spectrum acquisition was performed in positive‐ion mode over *m/z* 190–1500 using MS^E^ under gentle conditions (0 V collision potential, function 1) and fragmenting conditions (collision energy ramp 20–80 V, function 2), with scan time of 0.2 s. Leucine enkephalin [M+H]^+^ was used as lock mass, with its signal sampled every 10 s. Accurate masses and fragments were confirmed in UHPLC/MS/MS (positive‐ion mode, *m/z* 50–1500, collision energy ramp 20–80 V).

### Subcellular localisation *P. vulgaris* TPSs

GFP‐fused constructs were prepared by cloning the full‐length coding sequences of *PvTPS2*,* PvHVS*,* PvTPS4* and *PvTPS5* into pEAQ_HT_GFP vector (kindly provided by Prof. G. Lomonossoff, John Innes Centre, UK) to create C‐terminal GFP tagged PvTPS constructs namely pEAQ_PvTPS2::GFP, pEAQ_PvHVS::GFP, pEAQ_PvTPS4::GFP and pEAQ_PvTPS5::GFP. Sequence verified constructs were transformed into the LBA4404 *A. tumefaciens* strain by electroporation. Transient expression assays in 4‐wk‐old *N. benthamiana* leaves were conducted as mentioned above. Fluorescence of the fusion proteins was observed 5 d after agroinfiltration, using a Fluoview FV10i confocal laser‐scanning microscope (Olympus Corporation, Tokyo, Japan).

### Homology modelling

Homology models for each enzyme were made using i‐tasser (v.5.1) (Roy *et al*., 2010; Yang *et al*., 2015) with epi‐aristolochene synthase from *Nicotiana tabacum* (Starks *et al*., 1997) (PDB ID: 5EAU) as the template structure. Position‐specific scoring matrices (PSSMs) were generated for both plastidial enzymes with two iterations of Psi‐Blast (Altschul *et al*., 2009) against the nonredundant protein sequences database. Active site positions were determined following a structural alignment between each homology model and the template structure and finding each position where any homology model had a residue within 4 Å of the ligands bound in the template. As only *c*. 5% of TPS‐a have a plastidial targeting sequence, the PSSM scores largely reflect cytosolic enzymes. Distal positions of interest were chosen by finding residues for which the PSSM score was at least by 9 units lower than any other residue at that position in both PvHVS and PvTPS2, or where both PvHVS and PvTPS2 lost a proline in comparison with PvTPS4 and PvTPS5. Both active site and distal positions were narrowed down to positions where residues were chemically similar within both cytosolic and both plastidial enzymes, but chemically different between each type. Eleven positions were identified that met these criteria and are highlighted in homology models shown in Fig. S1. These positions are also highlighted in a multiple sequence alignment made with clustalomega (v.1.2.4) (Sievers *et al*., 2011), which also includes examples of both cytosolic and plastidial TPS‐a enzymes from Euphorbiaceae, Solanaceae, and Brassicaceae (Fig. S2).

### Broad transcriptome and genome screening for plastidial TPS‐a enzymes

To estimate how widespread TPS‐a compartment switching is among plants, we first downloaded all known available plant transcriptome assemblies: Medicinal plant sequencing projects (http://medicinalplantgenomics.msu.edu/, https://apps.pharmacy.uic.edu/depts/pcrps/MedTranscriptomePlants/, and https://bioinformatics.tugraz.at/phytometasyn/), the NCBI Transcriptome Shotgun Assembly archive (TSA) (Benson *et al*., 2013), the Mint Evolutionary Genomics Consortium (http://mints.plantbiology.msu.edu/index.html), and gene models from ensembl plants (v.37) (https://plants.ensembl.org/index.html) (Kersey *et al*., 2018). We annotated each of the transcriptomes and searched for TPS candidate genes as described above for the *P. vulgaris* transcriptomes, except that a cutoff 70% coverage to the closest reference sequence was used. A candidate was classified as TPS‐a if the closest reference sequence was a TPS‐a, and the identity was at least 40%. Candidates were counted as ‘plastidial’ if they were predicted by targetp, with reliability class 3 or better, to be targeted to the plastid.

## Results

### Identifying candidate TPS genes

**Figure 3:**
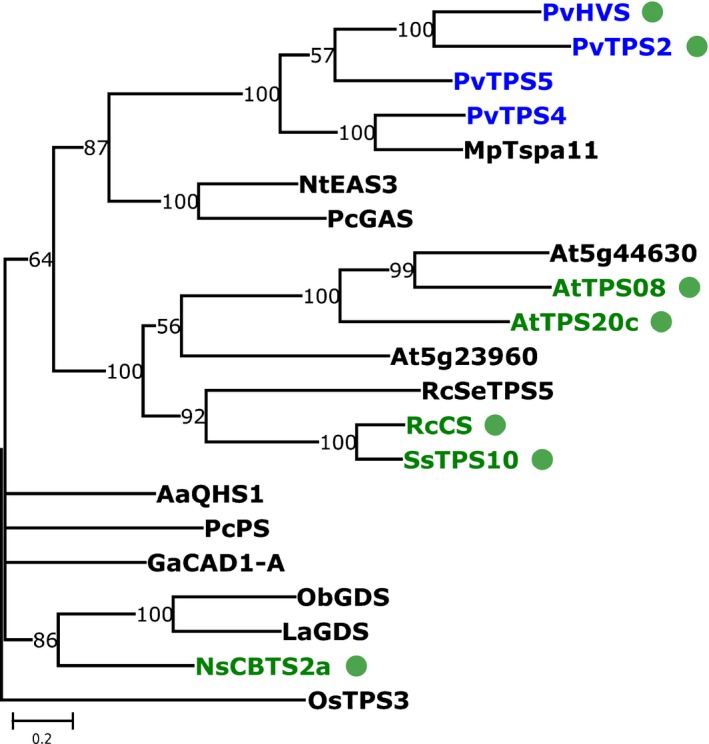
Maximum likelihood tree of selected TPS‐a subfamily peptide sequences. Green dots indicate a sequence with a plastid transit peptide. Green names are diterpene synthases, black are sesquiterpene synthases, blue are the four new clones from *Prunella vulgaris*. The tree is rooted to OsTPS3, a TPS‐a gene from the monocot rice (*Oryza sativa*). Scale bar indicates substitutions per site. Numbers at internal nodes indicate percent bootstrap support. Branches with < 50% support have been collapsed. Species abbreviations: Aa, *Artemisia annua*; At, *Arabidopsis thaliana*; Ga, *Gossypium arboreum*; La, *Lavandula angustifolia*; Mp, *Mentha x piperita*; Ns, *Nicotiana sylvestris*; Nt, *Nicotiana tabacum*; Ob, *Ocimum basilicum*; Os, *Oryza sativa*; Pc, *Pogostemon cablin*; Pv, *Prunella vulgaris;* Rc, *Ricinus communis*; Ss, *Sapium sebiferum*. Full sequences, alignment and methods for the tree can be found in Supporting Information Dataset S3.

We used Blast searches (Camacho *et al*., 2009) against a set of reference sequences to identify candidate TPS genes from the root and leaf transcriptomes. Blast results from *P. vulgaris* showed 18 TPS candidate sequences in the roots, and 10 in the leaves (Table 1; Dataset S2). Subcellular localisation prediction revealed that certain individual TPS‐a sequences in the roots contained plastid transit peptides. In plants, (*E*,*E*)‐FPP used in specialised metabolism is thought to derive from the cytosolic mevalonate pathway, and GGPP used in specialised metabolism is thought to derive from the plastidial methylerythritol 4‐phosphate pathway (Tholl, 2015). Most enzymes from the TPS‐a subfamily have been identified as cytosolic sesquiterpene synthases, using (*E*,*E*)‐FPP as a substrate. However, TPS‐a enzymes localised to the plastid and utilising GGPP have been implicated in the formation of nonlabdane‐related diterpene skeletons in several angiosperm lineages (Mau & West, 1994; Ennajdaoui *et al*., 2010; Wang *et al*., 2016). The four most highly expressed TPS transcripts in the root encode enzymes from the TPS‐a subfamily, among them, two had predicted transit peptides. This finding led us to select these four genes for cloning and functional characterisation. A maximum likelihood tree including the amino acid sequences of the four *P. vulgaris* TPS candidates together with those of selected TPS‐a enzymes from the literature (Fig. 3) suggests that the *P. vulgaris* TPS enzymes are most closely related to cytosolic sesquiterpene synthases from Lamiaceae and Solanaceae. In the phylogenetic tree, all the cytosolic reference sequences have demonstrated sesquiTPS activity and all the putatively plastidial sequences have demonstrated diTPS activity.

**Table 1:** Summary of TPS candidates from *Prunella vulgaris* root and leaf transcriptomes

	Root	Leaf
TPS‐a	7 (3)	6
TPS‐b	4	1
TPS‐c	1 (1)	0
TPS‐e	3 (2)	1 (1)
TPS‐f	1	1
TPS‐g	2	0
Total	18	10

Number in parentheses indicates the number of these candidates predicted to have transit peptides (targetp reliability class 3 or better). For example, there were seven candidate TPS‐a genes from root, three of them have putative plastidial transit peptides. The TPS sets from leaf and root are mostly nonoverlapping, with only three nearly identical pairs between the two datasets. Within each assembly there is some possible redundancy arising from the choice to include all isoforms of each transcript, rather than try to select a representative isoform.

A *de novo* assembly of newly generated Oxford Nanopore root RNA‐seq data did not lead to the identification of additional candidate TPS genes. However, the reads and assembly are made available.

### Enzyme activity and substrate specificity

**Figure 4:**
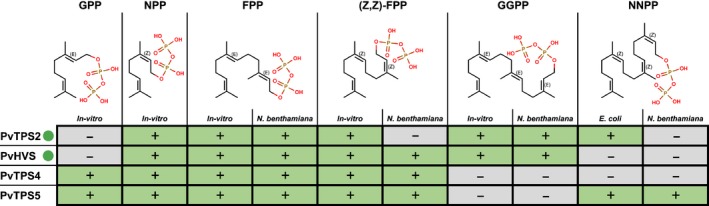
Summary of activity assays. TPSs were assayed *in vitro* with a range of prenyl‐diphosphates and through co‐expression with prenyl‐diphosphate synthases in *Nicotiana benthamiana* or *Escherichia coli*. Green box with ‘+’, activity detected; Grey box with ‘−’, activity not detected. Green dots by sequence names indicate enzymes that are natively localised to the plastid. FPP, farnesyl diphosphate; GGPP, geranylgeranyl diphosphate; GPP, geranyl diphosphate; NNPP, nerylneryl diphosphate; NPP, neryl diphosphate.

The activities of the four *P. vulgaris* TPS candidates were investigated through *in vitro* assays and expression in *E. coli* and *N. benthamiana* (Fig. 4). For *in vitro* and *E. coli* expression assays, recombinant enzymes were produced in *E. coli*: PvHVS, PvTPS2, and NNPP synthase (which natively include an N‐terminal transit peptide) were produced as truncated proteins lacking the predicted transit peptide, while PvTPS3 and PvTPS4 (which natively lack a transit peptide) were produced in their full‐length form. Recombinant proteins were either purified from the cell lysate for *in vitro* assays or produced with upstream pathway enzymes to produce terpenoids *in vivo*. For transient expression assays in *N. benthamiana*, PvTPS4, PvTPS5, and (*E*,*E*)‐FPP synthase (which natively lack a transit peptide) were produced fused to an N‐terminal plastid transit peptide from *A. thaliana* RubisCO small subunit; all other enzymes were predicted to contain a plastid transit peptide and were produced as full‐length proteins. Terpene compounds were identified in hexane extracts through GC‐MS analysis (Fig. S3). In most cases, the products were tentatively annotated based on comparisons of mass spectra to the Adams (2009) or NIST17 mass spectral databases (Fig. S4). We use bold letters in parentheses to indicate corresponding peaks and chromatograms from Figs S3 and S4.

All four enzymes were active against (*E*,*E*)‐FPP both *in vitro*, and in *N. benthamiana*. The major products varied substantially among the enzymes. PvTPS2 produced acyclic sesquiterpene alkenes (g, h, i) and alcohols (j). PvHVS produced a product with a mass spectrum resembling bisabolol (k) in *N. benthamiana*, and a mix of putative bisabolol (k) and farnesene (g) *in vitro*. *In vitro*, PvTPS4 catalysed the formation of a product annotated as the bicyclic sesquiterpene δ‐cadinene (l), while PvTPS5 products were annotated as tricyclic sesquiterpenes such as β‐barbatene (o). The *in vitro* products of PvTPS5 from (*E*,*E*)‐FPP were consistent with the major sesquiterpene peaks observed in *P. vulgaris* root extract (Fig. S3, peaks m, n, and o).

**Figure 5:**
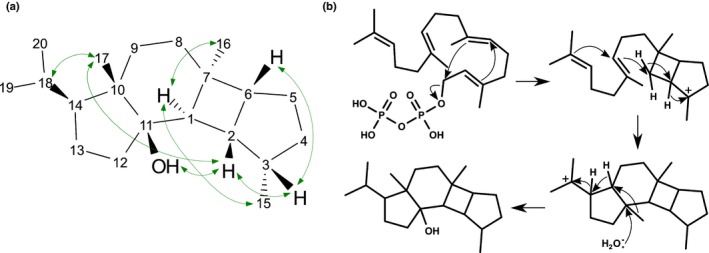
Product structure and proposed mechanism of reaction catalysed by PvHVS from geranylgeranyl diphosphate (GGPP). (a) Product determined by NMR to be 11‐hydroxy vulgarisane. Green arrows indicate NOESY correlations supporting the assignment of relative stereochemistry. (b) Possible mechanism for cyclisation of GGPP into 11‐hydroxy vulgarisane, as previously proposed (Lou *et al*., 2014).

In *N. benthamiana*, PvTPS4 and PvTPS5 produced a major product (q) from (*E*,*E*)‐FPP, which had no close hits in the mass spectral databases. Because this product was absent from the *in vitro* assays, we sought to determine if this product was truly a derivative of (*E*,*E*)‐FPP, rather than of an endogenous substrate in the *N. benthamiana* plastid. We expressed all four TPSs under two additional conditions: without co‐expression of a prenyl‐transferase, and with co‐expression of a (*Z*,*Z*)‐FPP synthase, an enzyme known to be present in the plastid in certain members of the Solanaceae (Sallaud *et al*., 2009). The results indicated that the new product only occurred in *N. benthamiana* when PvTPS4 and PvTSP5 were co‐produced with (*E*,*E*)‐FPP synthase (Fig. S5). *In vitro*, all four TPSs were active against (*Z*,*Z*)‐FPP, with the major products annotated as monocyclic sesquiterpenes such as bisabolene (t, u), except for PvTPS4 whose major product (s) did not have a close match in the spectral databases. Only PvTPS2 and PvHVS were active *in vitro* and in *N. benthamiana* against GGPP. PvTPS2 produced small amounts of an unidentified diterpene (x). Assays with PvHVS produced small amounts of a different unidentified diterpene (y), as well as large amounts of 11‐hydroxy vulgarisane (z). Low accumulation of diterpenes x and y in both *N. benthamiana* and *in vitro* assays precluded purification for structural elucidation by NMR and possibly masked the parental ion. The minor diterpene product of PvHVS (y) was observed by GC‐MS of *P. vulgaris* root extract. This product may be a metabolic dead‐end, whereas the major product, 11‐hydroxy vulgarisane may be quickly turned over along the pathway to the vulgarisins. The structure of 11‐hydroxy vulgarisane was confirmed by a series of NMR experiments on the purified product (Figs 5a, S6), with the NOESY spectrum supporting a relative stereochemistry assignment consistent with that reported for vulgarisins A–D originally reported from whole‐plant extracts (Lou *et al*., 2014, 2017). A possible mechanism for the cyclisation of GGPP into 11‐hydroxy vulgarisane is shown in Fig. 5(b). Our transcriptome analyses and enzyme activity assays suggested the roots as the likely site of vulgarisin biosynthesis. While we did not detect 11‐hydroxy vulgarisin in root extracts by GC‐MS, UHPLC/MS^E^ analysis found 11‐hydroxy vulgarisane (Fig. S7; Table S2) as well as two vulgarisin A and D isomers (Fig. S8; Table S2) in root extracts, but not in leaf extracts.

Upon observing weak activity from PvTPS2 with the unexpected substrate (*Z*,*Z*)‐FPP, and not observing strong activity from PvTPS2 against any substrate, we decided to assay all enzymes with additional substrates to test the extent of their promiscuity, and to try to find a preferred substrate for PvTPS2. Activities against GPP and NPP substrates were tested *in vitro*. Two enzymes, PvTPS4 and PvTPS5 were found to use GPP as a substrate, resulting in mixtures of products including limonene (c). By contrast, all four enzymes were found to catalyse cyclisation of NPP, with the major product being limonene, except for PvTPS4, which made more α‐terpinene (b) than limonene. Limonene was identified by comparison with an authentic standard. As NNPP is not commercially available, we tested this substrate by co‐expressing NNPP synthase with the TPSs in *E. coli* and *N. benthamiana*. PvTPS5 was active against NNPP in both systems, producing an unidentified product (aa). Trace amounts of the same compound were observed from *E. coli* co‐expressing PvTPS2 with NNPP synthase.

To investigate whether differences in substrate specificity could be associated with changes of amino acid residues at specific positions, we compared homology models of the four *P. vulgaris* enzymes (Fig. S1), and sequence alignments of representative cytosolic and plastidial TPS‐a enzymes from four different plant families (Fig. S2). Beyond the presence of plastidial transit peptides, we were unable to identify any sequence features consistently differing between the diterpene synthases and the sesquiterpene synthases.

### Subcellular localisation of PvTPS::GFP fusion proteins

**Figure 6:**
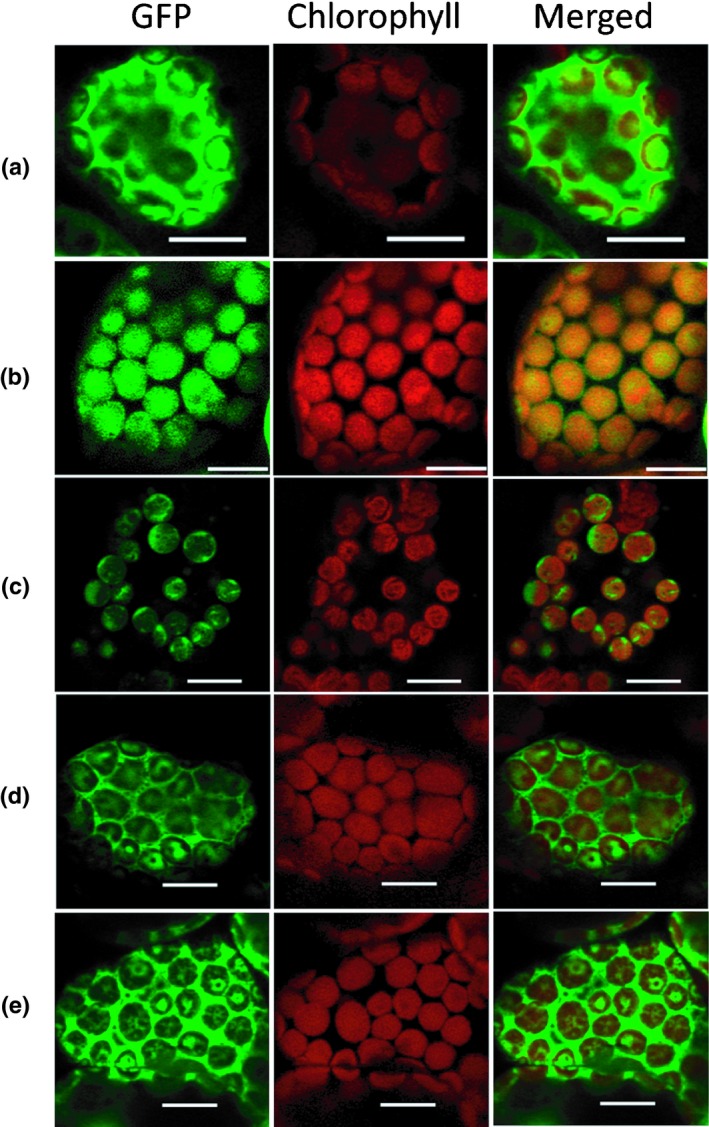
Transient expression of *Prunella vulgaris* TPSs in tobacco leaf epidermal cells and subcellular localisation by confocal laser‐scanning microscopy. (a) Cytoplasmic localisation of 35S::GFP vector control. (b, c) Plastidic localisation of PvTPS2::GFP and PvHVS::GFP, respectively. (d, e) Cytoplasmic localisation of PvTPS4::GFP and PvTPS5::GFP, respectively. GFP fluorescence (green), chlorophyll autofluorescence (red) and merged images (green and red) are shown. Bars, 10 μm.

To experimentally verify the targetp predictions and to corroborate the biochemical validation of *P. vulgaris* TPSs, each TPS was fused in‐frame to GFP. The resultant chimeric protein was transiently expressed in 4‐wk‐old tobacco leaf epidermal cells followed by investigation by confocal laser‐scanning microscopy. pEAQ_HT_GFP showed a typical nuclear and cytoplasmic localisation for GFP (Fig. 6a), while PvTPS2::GFP (Fig. 6b) and PvHVS::GFP (Fig. 6c) were targeted to plastids as evidenced by the overlap of GFP signal with the red chloroplast autofluorescence. Conversely, PvTPS4::GFP (Fig. 6d) and PvTPS5::GFP(Fig. 6e) showed expression patterns resembling that of pEAQ_HT_GFP, indicating that they are also targeted the cytosol.

### Prevalence of compartment switching among plant TPS enzymes

To more systematically assess the evidence for substrate and compartment switching as driving forces in TPS evolution, we searched the literature for examples of angiosperm TPSs with noncanonical substrates or subcellular localisation. We found 22 examples of these anomalous TPSs (Table 2). Focusing just on the TPS‐a subfamily we mined publicly available transcriptomes and gene models for sequences with putative transit peptides and found sequences encoding putative plastid transit peptides in *c*. 5% of TPS‐a genes (Table 3).

**Table 2:** Examples of angiosperm TPSs which have localisation or substrate specificity different from what might be expected based on the TPS subfamily

Name	Subfamily	Species (Family)	Plastidial	Major substrate	UniProt	Reference
AtTPS08	TPS‐a	*Arabidopsis thaliana* (Brassicaceae)	**Yes**	**GGPP**	O65435	Vaughan *et al*. (2013)
AtTPS18	TPS‐a	*Arabidopsis thaliana* (Brassicaceae)	**Yes**	**GFPP**	Q9LUE2	Shao *et al*. (2017)
AtTPS19	TPS‐a	*Arabidopsis thaliana* (Brassicaceae)	**Yes**	**GFPP**	Q9LUE0	Shao *et al*. (2017)
AtTPS20c	TPS‐a	*Arabidopsis thaliana* (Brassicaceae)	**Yes**	**GGPP**	A0A178U9Y5	Wang *et al*. (2016)
AtTPS25	TPS‐a	*Arabidopsis thaliana* (Brassicaceae)	**Yes**	**GFPP**	Q9LIA1	Huang *et al*. (2017)
AtTPS30	TPS‐a	*Arabidopsis thaliana* (Brassicaceae)	**Yes**	**GFPP**	Q9LH31	Huang *et al*. (2017)
Bo250	TPS‐a	*Brassica oleracea* (Brassicaceae)	**Yes**	**GFPP**	A0A0D3CK74	Huang *et al*. (2017)
Cr237	TPS‐a	*Capsella rubella* (Brassicaceae)	**Yes**	**GFPP**	R0GB30	Huang *et al*. (2017)
FvPINS	TPS‐a	Fregaria vesca (Rosaceae)	No	**GPP**	O23945	Aharoni *et al*. (2004)
NsCBTS2a	TPS‐a	*Nicotiana sylvestris* (Solanaceae)	**Yes**	**GGPP**	D9J0D3	Ennajdaoui *et al*. (2010)
PvHVS	TPS‐a	*Prunella vulgaris* (Lamiaceae)	**Yes**	**GGPP**		This work
ZmSTC1	TPS‐a	*Zea mays* (Poaceae)	**Yes**	**GPP**	Q7FU79	Lin *et al*. (2008)
ZmTPS26	TPS‐a	*Zea mays* (Poaceae)	**Yes**	**GPP**	A5YZT3	Lin *et al*. (2008)
ArTPS03	TPS‐b	*Arabidopsis thaliana* (Brassicaceae)	**No**	**(** ***E*** **,** ***E*** **)‐FPP**	A4FVP2	Huang *et al*. (2010)
ObZIS	TPS‐b	*Ocimum basilicum* (Lamiaceae)	**No**	**(** ***E*** **,** ***E*** **)‐FPP**	Q5SBP4	Iijima *et al*. (2004)
TrTPS8	TPS‐b	*Tripterygium regelii* (Celastraceae)	Yes	**CPP**	A0A222G0T8	Inabuy *et al*. (2017)
TwTPS27	TPS‐b	*Tripterygium wilfordii* (Celastraceae)	Yes	**CPP**	A0A1C7AAM8	Hansen *et al*. (2017)
CbLIN	TPS‐e/f	*Clarkia breweri* (Onagraceae)	Uncertain	**GPP**	Q96376	Cseke *et al*. (1998)
ShSBS	TPS‐e/f	*Solanum habrochaites* (Solanaceae)	Yes	**(** ***Z*** **,** ***Z*** **)‐FPP**	B8XA41	Sallaud *et al*. (2009)
SlPHS1	TPS‐e/f	*Solanum lycopersicum* (Solanaceae)	Yes	**NPP**	C1K5M3	Schilmiller *et al*. (2009)
SlTPS21	TPS‐e/f	*Solanum lycopersicum* (Solanaceae)	Yes	**NNPP**	G5CV51	Matsuba *et al*. (2013)
TaKSL5	TPS‐e/f	*Triticum aestivum* (Poaceae)	Yes	**(** ***E*** **,** ***E*** **)‐FPP**	G9M5S8	Hillwig *et al*. (2011)
ZmTPS1	TPS‐e/f	*Zea mays* (Poaceae)	**No**	**(** ***E*** **,** ***E*** **)‐FPP**	Q84ZW8	Schnee *et al*. (2002)

Bold indicates exceptions to what is typical for a subfamily. Plastidial TPS‐a diTPSs from Euphorbiacae are omitted because they are well established in the literature.

CPP, copalyl diphosphate; FPP, farnesyl diphosphate; GFPP, geranylfarnesyl diphosphate; GGPP, geranylgeranyl diphosphate; GPP, geranyl diphosphate; NNPP, nerylneryl diphosphate; NPP, neryl diphosphate.

**Table 3:** Frequency of plastidial transit peptides in TPS‐a genes from publicly available plant transcriptome data

	Total TPSs	TPS‐a	Plastidial TPS‐a
Medicinal plants projects	1911	637	32
NCBI‐TSA	4024	1000	44
Mint Evolutionary Genomics Consortium	903	221	3
EnsemblPlants v.37	1610	430	38
Total	8448	2288	117

Some plants were present in more than one of the datasets, so the rows are not entirely independent.

## Discussion

### Implications for biosynthesis of terpenoids in *P. vulgaris* roots

We found that PvHVS catalyses the reaction consistent with the first step in the previously proposed vulgarisin pathway (Lou *et al*., 2014), the formation of 11‐hydroxy vulgarisane. The remaining steps are likely to be catalysed by one or more cytochromes P450, isobutyl transferases, and benzoyl transferases. We detected 11‐hydroxy vulgarisane, as well as isomers of vulgarisins A and D in root, but not in leaf extracts. However, we were unable to detect masses matching the calculated exact masses or predicted fragmentation of the proposed intermediates (Fig. 2), so the order of the modification steps remains unclear.

According to root RNA‐seq data, *PvTPS2* and *PvTPS5* are the most and second most highly expressed TPSs, respectively. We found that the ratio of PvTPS5 products from assays with (*E*,*E*)‐FPP, with the exception of two products (p and q) which were not detected in roots, resembled the sesquiterpene metabolite pattern observed in *P. vulgaris* root extract, suggesting that native PvTPS5 functions as a major sesquiterpene synthase in roots. Even though *PvTPS2* is the most highly expressed TPS gene in *P. vulgaris* root tissue and is active against prenyl‐diphosphate substrates of multiple chain lengths, we were unable to connect any of the products of assays with recombinant PvTPS2 to metabolites detected in root extracts. It is possible that the preferred substrate of PvTPS2 has an unusual chain length or double‐bond configuration that was not among the prenyl‐diphosphate substrates tested in this study. It is also possible that the products of PvTPS2 are rapidly turned over and do not accumulate.

### Compartment and substrate switching among plant TPSs

PvHVS is the first reported diterpene synthase in the TPS‐a subfamily from Lamiaceae. Based on phylogenetic analysis (Fig. 3) it seems that cytosolic sesquiTPSs from TPS‐a acquired plastid transit peptides and diTPS activity independently in at least four different angiosperm lineages, Euphorbiaceae (Mau & West, 1994), Solanaceae (Ennajdaoui *et al*., 2010), Brassicaceae (Vaughan *et al*., 2013), and now Lamiaceae. Given that TPSs have been characterised from only a small fraction of angiosperm families, it is plausible that investigation of additional enzymes from TPS‐a will reveal further independent instances of substrate and compartment switching. Within Lamiaceae, there are reports of nonlabdane‐related diterpenes from other species, for example cembranoid diterpenes from *Anisomeles indica* (Chen *et al*., 2008) and *Isodon sculponeatus* (Li *et al*., 2009), and the unusual diterpenoids from *Salvia sclarea* and *Leucosceptrum canum* (Fig. 1) (Laville *et al*., 2012; Luo *et al*., 2012). Determining whether the cyclisation of these diterpenes is catalysed by plastidial TPS‐a enzymes and, if so, whether those enzymes are encoded by orthologs of *PvHVS* would help to answer the question of how rare of an event a substrate or compartment change is for a TPS, and whether it occurred multiple times within Lamiaceae.

It appears that a change of compartment occurred at least three times in the evolutionary histories each individual plastidial TPS‐a enzyme. One plausible evolutionary model is as follows: first, sometime between the endosymbotic origin of the plastid and the divergence of land plants, an ancestor or early form of a bifunctional *ent*‐copalyl diphosphate/*ent*‐kaurene synthase acquired a transit peptide. Second, after loss of the class II function, the TPS lost its transit peptide and evolved to become a monofunctional cytosolic sesquiTPS, the founder of the TPS‐a subfamily. Finally, a TPS‐a reacquired a plastid‐targeting sequence and evolved into a diTPS once again, which appears to have happened independently in multiple lineages. Other examples of parallel evolution in TPSs are the gain and loss of transit peptides in the gymnosperm TPS‐d subfamily (Martin *et al*., 2004), and the loss of the γ‐domain (Hillwig *et al*., 2011), which also seem to have occurred multiple times independently during TPS evolution.

A recent review found 40 multisubstrate plant TPSs (Pazouki & Niinemets, 2016), providing strong evidence that promiscuous TPSs are prevalent within the plant kingdom. Combined with our own literature search, we found 22 examples of angiosperm TPSs with unexpected localisation or preferred substrate (Table 2). This approach raises the possibility that an inherent lack of substrate specificity in some TPSs may play a role in their evolution. In this model, TPSs are subject to selective pressure for high activity against a substrate available in the subcellular compartment, but activity against other substrates, particularly substrates not occurring in the compartment, may not be selected against. In this model it is expected that only a few mutations, possibly coinciding with acquisition or loss of a transit peptide, would suffice to alter a TPS's preferred substrate, thereby giving the plant access to a novel specialised metabolite that may convey some selective advantage and lead to the activity becoming fixed in the population.

The results of our characterisation of four closely related TPS‐a enzymes from *P. vulgaris* are consistent with this model of TPS evolution. All four enzymes, even the two plastidial enzymes, showed activity with (*E*,*E*)‐FPP, the presumed substrate of the common ancestral protein. Furthermore, all enzymes showed some degree of activity on unusual substrates such as NPP, (*Z*,*Z*)‐FPP, or NNPP, indicating an ability to act on substrates that they do not normally encounter. Additional support for this model of TPS evolution comes from an alignment of TPS‐a diTPSs arising from the four known independent substrate switching events (Fig. S2). The TPSs show no common signature, suggesting that there is not one specific mutation or set of mutations responsible for the change in substrate specificity, and opening the possibility that a wide variety of mutations could result in substrate switching, and obviating the need for a specific low‐probability event.

Yet more evidence for our proposed model comes from the frequent co‐occurrence of TPS substrate and compartment switching (Table 2). To name just a few examples: maize STC1 and TPS26 are plastidial TPS‐a monoterpene synthases (Lin *et al*., 2008), and basil ObZIS is a cytosolic TPS‐b sesquiterpene synthase (Iijima *et al*., 2004). Two of the more surprising recent developments were the discovery of a number of TPS‐a sesterterpene (C‐25) synthases in Brassicaceae (Huang *et al*., 2017; Shao *et al*., 2017), and the discovery of sesqui‐ and diTPSs in Solanaceae acting on *cis*‐prenyl‐diphosphates (Sallaud *et al*., 2009; Matsuba *et al*., 2013), together suggesting that there may be additional prenyl‐diphosphate substrates of unusual chain length or double‐bond configuration remaining to be discovered. A high degree of heterogeneity of TPS substrate preference and compartmentation has also been seen in the gymnosperms, where cytosolic sesquiterpene synthases seem to have arisen independently several times in the TPS‐d subfamily, which is dominated by plastidial monoTPSs and diTPSs (Martin *et al*., 2004; Mafu *et al*., 2017).

The results we report here pave the way for several possible new lines of inquiry. Open questions remain as to what sequence and structural changes in TPS‐a enzymes lead to changes in substrate preference and how likely those changes are to arise under conditions of random mutation and weak selection. It is now clear that screening TPS transcripts from genomic or transcriptomic data for putative transit peptides support recent changes to activity, and that one should not be too hasty in inferring substrate specificity based solely on TPS subfamily. Finally, the distinct product profiles from (*E*,*E*)‐FPP, with PvTPS2, PvHVS, PvTPS4, and PvTPS5 forming primarily acyclic, monocyclic, bicyclic, and tricyclic products, respectively, may make this set of enzymes a promising subject for studies to resolve which residues are important for product determination in sesquiterpene synthases.

Some earlier works have described TPSs with subcellular localisation or substrate usage that is different from the subfamily canonical norm as unusual, or unexpected. A growing body of evidence, including our characterisation of PvHVS, supports the hypothesis that changes in compartment or substrate specificity are common and widespread phenomena in the continuing evolution of plant TPSs.

## Author contributions

SRJ, WWB, RS, GPM and ASG performed the experiments and analysed the data. SRJ, RS, WWB and BH designed the experiments. SRJ conceived the project and wrote the initial manuscript draft. All authors edited the manuscript and approved the final version. WWB and RS contributed equally to this work.

## Supporting information

Please note: Wiley Blackwell are not responsible for the content or functionality of any Supporting Information supplied by the authors. Any queries (other than missing material) should be directed to the *New Phytologist* Central Office.

**Dataset S1** Tables of available Lamiaceae RNA‐seq datasets and the diterpene skeletons from those species.
**Dataset S2** Tables of candidate TPS genes from *P. vulgaris*.
**Dataset S3** Reference sequences and alignment for phylogenetic tree (Fig. 3).
**Fig. S1** Stereo view of homology models for each enzyme characterised in this study.
**Fig. S2** Multiple sequence alignment of cytosolic and plastidial TPS‐a enzymes found in Lamiaceae, Euphorbiaceae, Solanaceae, and Brassicaceae.
**Fig. S3** GC‐MS of hexane extracts TPS activity assays *in vitro*, in *E. coli*, and in *N. benthamiana*.
**Fig. S4** Mass spectra of enzyme products and selected reference spectra.
**Fig. S5** Peak q only appears when PvTPS4 or PvTPS5 is co‐expressed with (*E*,*E*)‐FPPS in *N. benthamiana*.
**Fig. S6** NMR spectra of the major product of PvHVS from GGPP, identified as 11‐hydroxy vulgarisane.
**Fig. S7** UHPLC/MS of 11‐Hydroxyvulgarisane in *P. vulgaris*.
**Fig. S8** UHPLC/MS of vulgarisin A and D isomers in *P. vulgaris*.
**Methods S1** Nanopore sequencing and assembly.
**Table S1** Exact masses and relative abundance for ions of vulgarisin‐related compounds detected by UHPLC/MS in *P. vulgaris* root extracts.
**Table S2** Synthetic oligonucleotides used in this study.

## Data Availability

GenBank accessions of clones: PvTPS2, MH926014; PvHVS, MH926015; PvTPS4, MH926016; PvTPS5, MH926017. SRA accession of Nanopore sequencing of *P. vulgaris* root transcriptome: PRJNA491730. Raw and processed NMR and GC‐MS data, the Nanopore assembly, and the full sequences for the putative TPSs summarised in Table 3 (see later) are archived in Zenodo (doi: 10.5281/zenodo.1467956). The ^13^C and ^1^H NMR spectra for 11‐hydroxy vulgarisane were submitted to NMRshiftDB2.
