# Supplementary material for: Promiscuous terpene synthases from Prunella vulgaris highlight the importance of substrate and compartment switching in terpene synthase evolution
Source: New Phytol. 2019 Apr 8;223(1):323–35. doi: 10.1111/nph.15778 (PMC6593445; doi:10.1111/nph.15778)
Supplement: Supplementary file 1 — Dataset S1 Tables of available Lamiaceae RNA‐seq datasets and the diterpene skeletons from those species. Dataset S2 Tables of candidate TPS genes from P. vulgaris. Dataset S3 Reference sequences and alignment for phylogenetic tree (Fig. 3). Fig. S1 Stereo view of homology models for each enzyme characterised in this study. Fig. S2 Multiple sequence alignment of cytosolic and plastidial TPS‐a enzymes found in Lamiaceae, Euphorbiaceae, Solanaceae, and Brassicaceae. Fig. S3 GC‐MS of hexane extracts TPS activity assays in vitro, in E. coli, and in N. benthamiana. Fig. S4 Mass spectra of enzyme products and selected reference spectra. Fig. S5 Peak q only appears when PvTPS4 or PvTPS5 is co‐expressed with (E,E)‐FPPS in N. benthamiana. Fig. S6 NMR spectra of the major product of PvHVS from GGPP, identified as 11‐hydroxy vulgarisane. Fig. S7 UHPLC/MS of 11‐Hydroxyvulgarisane in P. vulgaris. Fig. S8 UHPLC/MS of vulgarisin A and D isomers in P. vulgaris. Methods S1 Nanopore sequencing and assembly. Table S1 Exact masses and relative abundance for ions of vulgarisin‐related compounds detected by UHPLC/MS in P. vulgaris root extracts. Table S2 Synthetic oligonucleotides used in this study. [file NPH-223-323-s001.pdf]

***New Phytologist* Supporting Information**

Article title: Promiscuous terpene synthases from *Prunella vulgaris* highlight the importance of substrate and compartment switching in terpene synthase evolution
Authors: Sean R. Johnson, Wajid Waheed Bhat, Radin Sadre, Garret P. Miller, Alekzander Sky Garcia, Björn Hamberger
Article acceptance date: 22 February 2019

The following Supporting Information is available for this article:

Method S1 Nanopore sequencing and assembly

Table S1 Synthetic oligonucleotides used in this study

Fig. S1 Stereo view of homology models for each enzyme characterized in this study

Fig. S2 Multiple sequence alignment of cytosolic and plastidial TPS-a enzymes found in Lamiaceae, Euphorbiaceae, Solanaceae, and Brassicaceae

Fig. S3 GC-MS of hexane extracts TPS activity assays in vitro, in *E. coli*, and in *N. benthamiana*

Fig. S4 Mass spectra of enzyme products and selected reference spectra

Fig. S5 Peak q only appears when PvTPS4 or PvTPS5 is co-expressed with FPPS in *N. benthaminana*

Fig. S6 NMR spectra of PvHVS major product from GGPP, identified as 11-hydroxy vulgarisane.

Fig. S7 UHPLC/MS of 11-Hydroxyvulgarisane in *P. vulgaris*

Table S2 Exact masses and relative abundance for ions of vulgarisin-related compounds detected by UHPLC/MS in *P. vulgaris* root extracts

Fig. S8 UHPLC/MS of vulgarisin A and D isomers in *P. vulgaris*

Methods S1. Nanopore sequencing and assembly.

RNA was extracted from root tissue according to a previously described method (Hamberger *et al.*, 2011), mRNA was selected from 16 µg total RNA using Dynabeads Oligo (dT)25 (ThermoFisher Scientific) yielding 119 ng mRNA. The mRNA was converted to cDNA using the Oxford Nanopore cDNA-PCR Sequencing Kit (SQK-PCS108) and sequenced on the GridIon. The resulting library consisted of 2,146,282 reads with an average length of 735 bp, and an N50 of 817 bp. The Illumina reads were from PhytoMetSyn were cleaned and normalized using Bbnorm (sourceforge.net/projects/bbmap/), and the raw reads were subjected to error correction using LoRDEC (Salmela & Rivals, 2014) according to settings suggested for transcriptome data (Hoang *et al.*, 2017). A hybrid assembly using the corrected Nanopore reads and the Illumina reads was generated using IDP-denovo (Fu *et al.*, 2018). The resulting assembly seemed to be of much worse quality than the Illumina-only assembly and we did not pursue further analysis.

Table S1. Synthetic oligonucleotides used in this study. Lower case letters indicate vector overhangs to enable In-Fusion (Takara) cloning.

Name	Sequence
PvTPS2 F	GATGAGCTCTCTCACAAATCTCTACAG
PvTPS2 R	TGAAGAATCAAACTTGATATGGATTGAGC
PvHVS F	CTCAAAATGAGCTCTCTCTCAATTCCC
PvHVS R	GTCTGAATGGGGAGTCAAATATG
PvTPS4 F	ATGGGTGAAGAGAGCAGTGTAAG
PvTPS4 R	CAAGGGAATCAAATGAGAAGAGG
PvTPS5 F	GAAATGGCAGCTGCAGGC
PvTPS5 R	TCAGATGACAAGAGGATTGAGTAGGAG
PvTPS2 pEAQ Infusion F	ttctgcccaaattcgCGAATGAGCTCTCTCACAAATCTCTA
PvTPS2 pEAQ Infusion R	agttaaaggcctcgaTCAAACTTGATATGGATTGAGCAGC
PvHVS pEAQ Infusion F	ttctgcccaaattcgCGAATGAGCTCTCTCTCAATTCCCTT
PvHVS pEAQ Infusion R	agttaaaggcctcgaTCAAATATGTATAGGATTCATCAATAGAGCAG
PvTPS4 pEAQ-TP Infusion F	ggcggaagagttaacATGGGTGAAGAGAGCAGTGTA
PvTPS4 pEAQ-TP Infusion R	agttaaaggcctcgaTCAAATGAGAAGAGGATTAAGAAACAAAGC
PvTPS5 pEAQ-TP Infusion F	ggcggaagagttaacATGGCAGCTGCAGGCATTG
PvTPS5 pEAQ-TP Infusion R	agttaaaggcctcgaTCAGATGACAAGAGGATTGAGTAGG
AtRuBisCO small pEAQ Infusion F	ttctgcccaaattcgATGGCTTCCTCTATGCTCTCTTC
AtRuBisCO small pEAQ Infusion R	agttaaaggcctcgaGTTAACTCTTCCGCCGTTGC
pET28plus PvTPS2 Infusion F	gaaggagatataccatgTCAGCTGTGATGGTGGATCATGC
pET28plus PvTPS2 Infusion R	ggtggtggtggtgctcgaAAACTTGATATGGATTGAGCAGC
pET28plus PvHVS Infusion F	gaaggagatataccatgTCAGCTGTGATGGTGGAAGG
pET28plus PvHVS Infusion R	ggtggtggtggtgctcgaAAATATGTATAGGATTCATCAATAGAGC
pET28plus PvTPS4 Infusion F	gaaggagatataccATGGGTGAAGAGAGCAGTGTAAGT
pET28plus PvTPS4 Infusion R	ggtggtggtggtggtgctcgaAAATGAGAAGAGGATTAAG
pET28plus PvTPS5 Infusion F	gaaggagatataccATGGCAGCTGCAGGCATTG
pET28plus PvTPS5 Infusion R	ggtggtggtggtgctcgaAGATGACAAGAGGATTGAGTAGG
zFPPS pEAQ-HT F	ttctgcccaaattcgATGAGTTCTTTGGTTCTTCAATGTTG
zFPPS pEAQ-HT R	agttaaaggcctcgaTCAATATGTGTGTCCACCAAAACG
NNPPS pEAQ-HT Infusion F	ttctgcccaaattcgATGAACTCTTCAATAGTGTCTCAACAC
NNPPS pEAQ-HT Infusion R	agttaaaggcctcgaTCAATATGTGTGTCCACCAAAAC
NNPPS F	ATGAACTCTTCAATAGTGTCTCAAC
NNPPS R	TCAATATGTGTGTCCACCAAAACG
NNPPS pACYCDuet Infusion F	aaggagatatacatATGTCTGATCGTGGACTCAGC
NNPPS pACYCDuet Infusion R	ctttaccagactcgagTCAATATGTGTGTCCACCAAAAC
pEAQ_PvTPS2_GFP_For	attctgcccaaattcgATGAGCTCTCTCACAAATCTCT
pEAQ_PvTPS2_GFP_Rev	ctagtcataccggtcgcAACTTGATATGGATTGAGCAG
pEAQ_PvHVS_GFP_For	attctgcccaaattcgATGAGCTCTCTCTCAATTCC

pEAQ_PvHVS_GFP_Rev	ctagtcataccggtcgcAATATGTATAGGATTCATCAATA
pEAQ_PvTPS4_GFP_For	attctgcccaaattcgATGGGTGAAGAGAGCAGT
pEAQ_PvTPS4_GFP_Rev	ctagtcataccggtcgcAATGAGAAGAGGATTAAGAAAC
pEAQ_PvTPS5_GFP_For	attctgcccaaattcgATGGCAGCTGCAGGCATT
pEAQ_PvTPS5_GFP_Rev	ctagtcataccggtcgcGATGACAAGAGGATTGAGT

Fig. S1. Stereo view of homology models for each enzyme characterized in this study. Select positions are highlighted in red. PvHVS: blue; PvTPS2: cyan; PvTPS4: yellow; PvTPS5: tan.

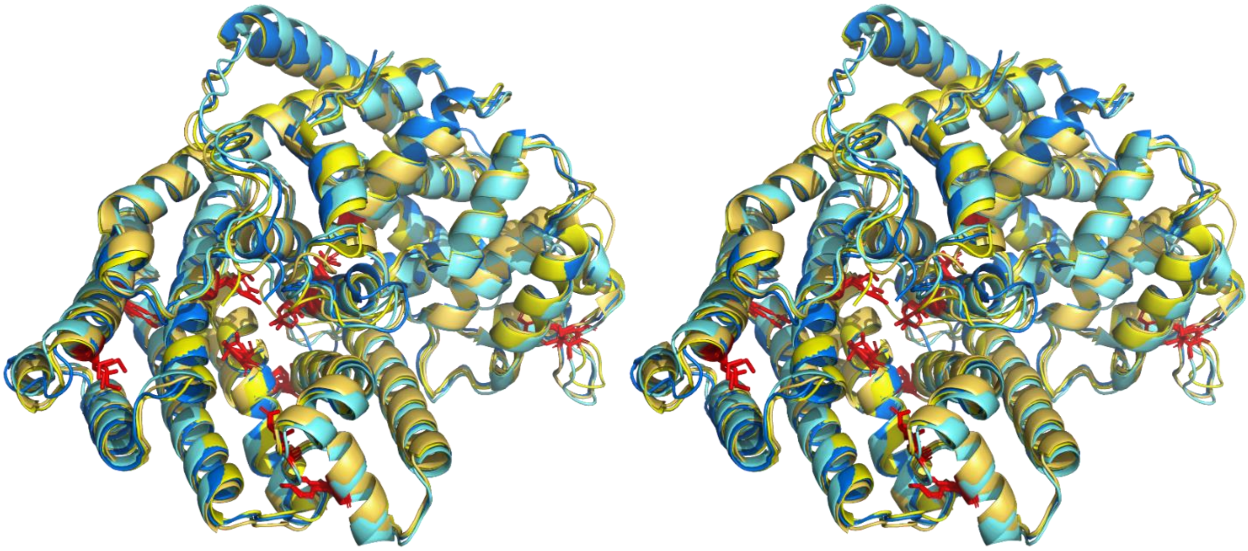

Fig. S2. Multiple sequence alignment of cytosolic and plastidial TPS-a enzymes found in Lamiaceae, Euphorbiaceae, Solanaceae, and Brassicaceae. Cytosolic and plastidial enzymes are above and below the central line, respectively, and enzymes from *P. vulgaris* characterized in this study are within the central box. Column highlights are explained in the main text. Accession numbers and families are indicated in the first line of the alignment. Abbreviations: RcAFS, *Ricinus communis* alpha-farnesene synthase; NtEAS, *Nicotiana tabacum* epi-aristolochene synthase; SlGDS, *Solanum lycopersicum* germacrene D synthase; AtTPS21/20, *Arabidopsis thaliana* terpene synthase 21/20; PvTPS2/4/5, *P. vulgaris* terpene synthase 2/4/5; PvHVS, *P. vulgaris* hydroxy vulgarisane synthase; RcCAS3, *Ricinus communis* casbene synthase; RcCAS2, *Ricinus communis* neocembrene synthase; NsCBTS3, *Nicotiana sylvestris* cembratrienol synthase 3.

```
AEQ27768.1      RcAFS   -----------------------------MKVGQPVLQCQTNS----------------- 14 ← Euphorbiaceae
5eau            NtEAS   ------------------------------------------------------------ 0  ← Solanaceae
XP_019070211.1  SlGDS   --------------------------------------------------MV-----VGF 5  ← Solanaceae
AAO85539.1      AtTPS21 ------------------------------------------------------------ 0  ← Brassicaceae
AZB50512.1      PvTPS4  ---------------------------------------------------------MG- 2  ← Lamiaceae
AZB50513.1      PvTPS5  ----------------------------------------------MAA-AG-----IVS 8  ← Lamiaceae Cytosolic
AZB50511.1      PvHVS   MSSLSIPFSSAICTSSIPKISTGHHRRT-----ARMPAHDTSRLV-FRPSAV-----MVE 49 ← Lamiaceae Plastidial
AZB50510.1      PvTPS2  MSSLTNLYSSIMTISTTKKVAVAHRLRTFNGHGRTVRRRTTRRLV-FRPSAV-----MVD 54 ← Lamiaceae
XM_002513297    RcCAS3  MALPPVAM-----QSNSEKLK-----YFFYRLPSTRLEYGNNRFH-FLPSSA-----KP- 43 ← Euphorbiaceae
XM_002513288    RcCAS2  MALQSLLFL----QANSQNRNF----CQFLSMPS--IRCCSCRVP-FSSWSA-----KS- 43 ← Euphorbiaceae
ADI87448.1      NsCBTS3 -------MS----QSI-SPLIC----SHFAKFQSNIWRCNTSQLR-VIHSSY-----ASF 38 ← Solanaceae
A0A178U9Y5.1    AtTPS20 MEA------------ITK----------NGSLSQTLVHCGPKSLSSFIPVRCLRFSKNPF 38 ← Brassicaceae

                RcAFS   -------------EAFGMMQERRSGNYK-PNIWKYDFLQSLSSKYDE-EKYKTQAERLKE 59
                NtEAS   -------MASAAVANYEEEIVRPVADFS-PSLWGDQFLSFSIDNQVA-EKYAKEIEALKE 51
                SlGDS   GGEAE--------------ITRRCANHH-PSVWGDHFLTYANLLGAN-EWEEKEHEDQKG 49
                AtTPS21 ---------------MGSEVNRPLADFP-ANIWEDPLTSFSKSDLGT-ETFKEKHSTLKE 43
                PvTPS4  ---------EESSVSCLRNSRPPVTDYV-PSIWADTFSTSSFKEQEQ-QKYEEAIEELKK 51
                PvTPS5  ----------AAVPTNIRQLRPPILKYK-PSMWGDTFATFSLDHQLQ-EKYSKEIETLKK 56
                PvHVS   G-------SPMTTSSNGKEVQRLITTFK-PSMWKDIFSTFSFDNQVQ-EKYLKEIEELKK 100
                PvTPS2  H-------AT--------EVRQVKTPYDPVSMWGNMFSTFSLDHQLQ-QKYSEEIEELKK 98
                RcCAS3  ---HAKPPAQACLSSTTHQEVRPLAHFP-PTVWGNRFASLTFNPSEF-ESYDGRVNVLKE 98
                RcCAS2  ---VTNKSPQACLSTKSQQEFRPLANFP-PTVWGSHFASPTFSESEF-GTYDRQANVLQK 98
                NsCBTS3 GGRRKERVRRMNRAMDLSSSSRHLADFP-STIWGDHFLSYNSEITEITTQEKNEHELLKE 97
                AtTPS20 PKKLVVTRARTSINSDHEAANRPLFQFP-PSLLDDRFLSISANQSEI-DSLGRDIEALKA 96

                RcAFS   DAKHLFIEA-VDLQGKLELVDCIIKVGLASHFKDEIKKALDTIASSI--KNDKSDAIKNR 116
                NtEAS   QTRNMLLATGMKLADTLNLIDTIERLGISYHFEKEIDDILDQIYNQN--S-----NCNDL 104
                SlGDS   GVRKMLVLSPSKSLQKLELINTIQLLGVSYHFEHEIEESLSEIYNGY--E-EWIGESHDL 106
                AtTPS21 AVKEAFMSSKANPIENIKFIDALCRLGVSCHFEKDIVEQLDKSFDCLDFPQMVRQEGCDL 103
                PvTPS4  EARCILLTAAASPRKQMILIDTLERLGLAYHFETEIEHILQQINQ-------QTLQDYDL 104
                PvTPS5  EVKSMLMAA--TSTQLMVLIDKLERLGLSYHFEAEIEDKLKQVYDL-----EEEVADHDL 109
                PvHVS   EVRSTLMSA--THRKLFDLIDNLERMGIAYHFETEIEDKLKQAHASL----EEE-DDYDL 153
                PvTPS2  NVRSTLMAA--TSTRLLMFIDNLERLGLAYHFETEIEDKLKQVYDSL----EKEGEDNDL 152
                RcCAS3  KVKDLLVSSATDSVETVVLIDLLCRLGVSYHFENDIEELLSKIFNSQ--PDLVDEKECDL 156
                RcCAS2  KIRELLTSSRSDSVEKIAFIDLLCRLGVSYHFENDIEEQLSQIFSCQ--PGLLDEKQYDL 156
                NsCBTS3 IVRKMLVETLDNSTQKLVLIDTIQRLGLAYHFNDEIENSIQNIFYLS--QNSEDNDEHNL 155
                AtTPS20 KVSEKLVCM--DVKERIHLIHLLVSLGVAYHFEKQIEEFLKVDFENV--E-DMNLGEEDM 151

                RcAFS   YVTALCFRLLRQHGYEVSQDVFSDFLDENGTFLKA---KSMDVKGVLELFEASYLALESE 173
                NtEAS   CTSALQFRLLRQHGFNISPEIFSKFQDENGKFKES---LASDVLGLLNLYEASHVRTHAD 161
                SlGDS   HDVALSFRLLRQQGYYVSSDVFRKFTDERGNYKET---LASDVQGLLSLYEAAQLRVDDE 163
                AtTPS21 YTVGIIFQVFRQFGFKLSADVFEKFKDENGKFKGH---LVTDAYGMLSLYEAAQWGTHGE 160
                PvTPS4  FTTTLGFRLLRQHRHHVSCSVFDKFLDQDAKFKESLL-LSSDTESVLSLYDAAHVRFRHE 163
                PvTPS5  FTTALRFRLLRQHQYHVSCDVFDKFVSKDGKWDLEISLSRDDIEGVLSLYEAAHVRIRDE 169
                PvHVS   FTTALRFRLLRQHRYHVSCDPFAKFVDQDNKLKES---LSSDVEGLLSLFEASHLRIHNE 210
                PvTPS2  FTTALRFRLLRQHRYQVSCDVFEQYFEEGKRVKES---HCSDVEGLLSLYEAAYVGIPNE 209
                RcCAS3  YTAAIVFRVFRQHGFKMSSDVFSKFKDSDGKFKES---LRGDAKGMLSLFEASHLSVHGE 213
                RcCAS2  YTVALVFRVFRQHGFKMSSNVFHKFTDSHGKFKAS---LLSDAKGMLSLFEASHLSMHGE 213
                NsCBTS3 YVAALRFRLARQQGNYMSSDVFKQFTNHDGKFKEN---HTNDVQGLLSLYEAAHMRVHDE 212
                AtTPS20 YSISVIFRVFRLYRHKLSSDVFNRFKEENGDFKKC---LLDDVRGMLSFYEASYFGTNTE 208
```

```
RcAFS     NILDDAKAFSTTILKDINSA-TTESNLYKQVVHALELPFHWRVRWFDVKWHIK-TFQKDK 231
NtEAS     DILEDALAFSTIHLESAAP--HLKSPLREQVTHALEQCLHKGVPRVETRFFISSIYDKEQ 219
SlGDS     EILDEAINFTTTHLKLLLP--NLSDPLATQVSNALKFPINNIIVRVATRKYIS-FYQEDK 220
AtTPS21   DIIDEALAFSRSHLEEISS--RSSPHLAIRIKNALKHPYHKGISRIETRQYISYYEEEES 218
PvTPS4    NLLKEAAVSTKQYLRGIEAE-LLDSSLKEKVNRALKHPLHRDVPIFYARFFIS-IYEKDL 221
PvTPS5    KVLDEAAAFTIHRLKHVLPQ-LESA-IKEKVELALQHPIHKSLPLLNFRSYIN-IYEGEG 226
PvHVS     DVLDEAIVFTTHHLNRMMPQ-LESP-LKEEVKHALRYPLHKCLGILSLRFHID-RYENDK 267
PvTPS2    KILDEAMTITTRQLTLMLPR-LESTILKEKVEHGLKFPVQRTNTIFSLRYYVE-IYENDE 267
RcCAS3    DILEEAFAFTKDYLQSSAVE--LFPNLKRHITNALEQPFHSGVPRLEARKFIDLYEADIE 271
RcCAS2    DILDEAFAFTKDYLESSAVDQYLCPNLQKHITNALEQPFHKGIPRLEARKYIDLYEGD-E 272
NsCBTS3   EILEEALIFTTTHLESVIP--NLSNSLKVQVTEALSHPIRKTIPRVGARKYIY-IYENIG 269
AtTPS20   EILDEAMGFTRKHLELFVGG-SNEEHLSGHIKNVLYLSQQENAEVVMSREYIQ-FYEQET 266

RcAFS     SINKTLLDLAKVNFNVVQATLQNDLKEISRWWRNLGLIENLK-FSRDRLVESFLCTVGLV 290
NtEAS     SKNNVLLRFAKLDFNLLQMLHKQELAQVSRWWKDLDFVTTLP-YARDRVVECYFWALGVY 278
SlGDS     SHNEMLLHFAKLDFNILQRLHKRELCDITRWWKDSEIAQALP-FARDRVVELYFWSLGVY 279
AtTPS21   C-DPTLLEFAKIDFNLLQILHREELACVTRWHHEMEFKSKVT-YTRHRITEAYLWSLGTY 276
PvTPS4    SRNELLLKLAKLNFNFLQNLYKEELFQLTGWWNKFDVKSKLT-YARDRLVEAYLWGVAYH 280
PvTPS5    LTNESVVKLSKFNFNFLQNIYRKELAELTSWWNKYELKSKLP-YARDRLVECYIWGAALR 285
PvHVS     SRDEVVLRLGQVNFNYMQNIYMNELYEITTWWNKLQMTSKVP-YFRDRLVECYMWGLAYH 326
PvTPS2    SRDALVLRLAKLNFNFMQNIYRSELSEATAWWNEYNLASEVP-YIRDRMVECYVSGVTYR 326
RcCAS3    CRNETLLEFAKLDYNRVQLLHQQELCQFSKWWKDLNLASDIP-YARDRMAEIFFWAVAMY 330
RcCAS2    CRNETVLEFAKLDYNRVQLLHQQELSQFSTWWKDLNLASEIP-YARDRMAEIFFWAVAMY 331
NsCBTS3   THNDLLLKFAKLDFNMLQKLHLKELNELTSWWKDLDCANKFP-YAKDRLVEAYFWTVGIY 328
AtTPS20   HHDETLLKFAKINFKFMQLHYVQELQTIVKWWKELDLESKIPNYYRVRAVECLYWAMAVY 326

RcAFS     FEPQYSSFRRWLTKVVIMILVIDDVYDIYGSLEELQHFTNAINRWDTAELEQ--LP-EYM 347
NtEAS     FEPQYSQARVMLVKTISMISIVDDTFDAYGTVKELEAYTDAIQRWDINEIDR--LP-DYM 335
SlGDS     FEPHYSVARKILTKVLCFCSIMDDTYDTYGTLDELTLLTTAIERWDIDASEQ--LP-SYM 336
AtTPS21   FEPQYSQARVITTMALILFTALDDMYDAYGTMEELELFTDAMDEWLPVVPDEIPIP-DSM 335
PvTPS4    YEPQYYNVRIGLVKGIQITGIMDDTYDNYATLNEAQIFTQTLDRWNANEVDG--LP-DYM 337
PvTPS5    YEPQFAYLRAIVAKTMQLVSIMDDTYDNYGTLEEDDLLTDILEKWNLDEIDV--LP-DFM 342
PvHVS     FEPEYAPVRVLITKYYMTATTVDDTYDNYATLEEIELFTQAIDRWSEDEIDQ--LPDEYL 384
PvTPS2    FEPQYSQIRMMVTKYYILGSVLDDTYDTYATPQEIDLMTQAMESWNTQETEK--LP-EYL 383
RcCAS3    FEPDYAHTRMIIAKVVLLISLIDDTIDAYATMEETHILAEAVARWDMSCLEK--LP-DYM 387
RcCAS2    FEPKYAQARMIIAKVVLLISLVDDTFDAYATIEETHLLAEAFERWDKSCLDQ--LP-DYM 388
NsCBTS3   FEPQYSRSRSMITKVVKMNSIIDDTYDAYATFDELVLFTDAIQRWDEGAMDS--LP-TYL 385
AtTPS20   MEPQYSVARIILSKSLVLWTIIDDLYDAYCTLPEAIAFTENMERWETDAKDM---P-DHM 382

RcAFS     KICFKTLHTITGETAHEMQREKRWDQEQTETHLKKVWADFCQALFVEAKWFNKGYTPSVQ 407
NtEAS     KISYKAILDLYKDYEKELSSAGR---SHIVCHAIERMKEVVRNYNVESTWFIEGYTPPVS 392
SlGDS     KLSYRALVQVYNETEKELENLGNKM-TYRVKYSINEMKKLLRAYFQEAKWYHGKDVPTME 395
AtTPS21   KFIYNVTVEFYDKLDEELEKEGR---SGCGFHLKKSLQKTANGYMQEAKWLKKDYIATFD 392
PvTPS4    KIVYDFILSIYEDYKCSASKHEK---GFSVPYFKEAVQELSSAYNQELEWVTERNMPSFI 394
PvTPS5    KIVYRFIMSVYEDFEREAEQQGK---SFAAPYYSESLKELSRAYNKEQKWIMERKMPPFE 399
PvHVS     KIVYKGLMNFTEEFRRDAEERGK---GYVIPYFIEETKRATQGYANEQRWIMKREMPSFE 441
PvTPS2    KLVLKAFMRFTEEFACEAEKQGK---SYVVPYVIEQMKEVGRRYAEAQEWTRAQESPTFQ 440
RcCAS3    KVIYKLLLNTFSEFEKELTAEGK---SYSVKYGREAFQELVRGYYLEAVWRDEGKIPSFD 444
RcCAS2    KVIYKLLLNTFSEFENDLAKEGK---SYSVRYGREAFQELVRGYYLEAMWRDEGKIPSFD 445
NsCBTS3   RSIYQGLLDVFNEMEEVLAKEGK---ADRIYYAKKEMKKLVAAYFKEAQWLNANYIPKCE 442
AtTPS20   KVLMRSFIDLHEDFKREVILEGR---LYSVEYGIDECKRLFREDLKLSKWARTGYIPNYD 439

RcAFS     EYLKTACISSSGSLLSVHSFFLIMNEGTREMLHFLEKNQE-MFYNISLIIRLCNDLGTSV 466
NtEAS     EYLSNALATTTYYYLATTSYLGMKS-ATEQDFEWLSKNPK-ILEASVIICRVIDDTATYE 450
SlGDS     QYIKNGIPSSTYLLLATTSWLGMGDVATKDAFDWISNEPT-ILVASSIIARLLNDLVTHE 454
AtTPS21   EYKENAILSSGYYGLIAMTFVRMTDVAKLDAFEWLNSHPK-IRVASEIISRFTDDISSYE 451
PvTPS4    EYARNSEITSSLYMMFAVIIPGLKS-LTPETIDWVKTEPL-ITVSTAMIGRYMDDIGSQH 452
PvTPS5    EYMTNSVFTSCIYPMFIAFVPGMKS-VTEKEVQWLLSEPK-IVISTAKMGRTLEDLGSHE 457
PvHVS     EYMVNSRVTSLMYVTYVAVVAVIES-ATKETVDWALSDSD-IFVYTNDIGRLIDDLATHR 499
PvTPS2    EYLANGRVTVLHYVLFPIIATFTKA-ATEETIHWLLDDSSKLVTWSADINRLLDDLATHQ 499
RcCAS3    DYLYNGSMTTGLPLVSTASFMGVQEITGLNEFQWLETNPK-LSYASGAFIRLVNDLTSHV 503
RcCAS2    EYIRNGSLSSGLPLVVTASFMGVKEITGIREFQWLRTKPK-LNHFSGAVGRIMNDIMSHV 504
NsCBTS3   EYMKNGVVTSTGTMYGIISLVVMEEIITKEAFEWLANEPL-ILRAASTICRLMDDMADHE 501
AtTPS20   EYMEVGIVTAGIDMTVAFAFIGMGE-AGKEAFDWIRSRPK-FIQTLDIKGRLRDDVATYK 497

RcAFS     AEQERGDAASSIVCHMREMEVLEEEARSYLKGIIGNYWKKVNEKCFTQSP--EMQLFINI 524
NtEAS     VEKSRGQIATGIECCMRDYGISTKEAMAKFQNMAETAWKDINEGLLRPTP--VSTEFLTP 508
SlGDS     IEVERGDVASGIECYMNEYGATKEEAYMEIRKIIENNWKDLNRGCLKPTT--VPRVLLMP 512
AtTPS21   FEHKREHVATGIDCYMQQFGVSKERAVEVMGNIVSDAWKDLNQELMRPHV--FPFPLLMR 509
PvTPS4    RESKGGKVLTAVDCYMKQYNVSKEETLSKFGELVEDAWEDLNKEWVETTS-FLPTEIAVQ 511
PvTPS5    RENRDGEMETVVDCYMKDKGVSKQRTIYEFEQLVENGWKDVTAEWAKENS--VCKEMVEH 515
PvHVS     RERKDGTMLTSMDYYMKEYGGTMEEGEAAFRKLMEEKWKLLNAAWVDTINGKESKEIVVQ 559
PvTPS2    RESKNGTVLTPMDFYMKEKGVSRQEAIIEVTKLLQETWKEINAEWVNCKT--VPKEICND 557
RcCAS3    TEQQRGHVASCIDCYMNQHGVSKDEAVKILQKMATDCWKEINEECMRRSQ--VSVGHLMR 561
RcCAS2    SEQNRGHVASCIDCYMKQYEVSKEEAIKEMQKMASDAWKDINEGYMRPAQ--VSVSELMR 562
NsCBTS3   VEQQREHVASFVECYMEEYGVSKQEAYVEMRKKITNACKDINKELLRPTA--VPMFILER 559
AtTPS20   DEMARGEIATGINCYMKQYKVTEEEAFLEFHRRIKHTSKLVNEEYFKTTV---PLKLVRI 554
```

```
RcAFS    NVNMARVVHNLY-QN-RDGFGVQDHQNKKQILSLLVHPFKLD-------------      564
NtEAS    ILNLARIVEVTYIHN-LDGYTHPEKVLKPHIINLLVDSIKI--------------      548
SlGDS    VLNLTRVAEF-FYKD-EDAYTFSKNNLKDVISMVLIDPIKA--------------      551
AtTPS21  VLNLSRVIDV-FYRY-QDAYTNPK-LLKEHIVSLLIETIPI--------------      547
PvTPS4   FLNYARMCDACYNSNDGDGYTDPAIFK-SNVVALFLNPLLI--------------      551
PvTPS5   LLNYGRIAEITYNNK-EDGYTEPEKYLGPLAAALLLNPLVI--------------      555
PvHVS    VLDLARICGTLYGDE-EDGFTYPEKNFAPLVAALLMNPIHI--------------      599
PvTPS2   LVEISRLSEIYYKSG-EDCYTNPEKNLAPIMAAVLLNPYQV--------------      597
RcCAS3   IVNLARLTDV-SYKY-GDGYTDSQ-QLKQFVKGLFVDPISI--------------      599
RcCAS2   VVNLARLTDV-SYKY-GDGYTDPQ-HLKQFVKGLFIDPVPLPNQIRKGETKTKHV      614
NsCBTS3  TLNFIRLVGT-FLKD-DDGYTNPKSQVKDSIALLFVESIDI--------------      598
AtTPS20  AFNVGRAIDTNY-KH-GDGLTYGG-IVEGQITSLFLDLITI--------------      592
```

Fig. S3. GC-MS of hexane extracts TPS activity assays *in vitro*, in *E. coli*, and in *N. benthamiana*. *In vitro*, purified enzymes were fed with pure precursors. For the *N. benthamiana* assays, DXS is overexpressed in all conditions. Chromatograms labeled *P. vulgaris* root are from methyl tert-butyl ether extracts of fresh root. Key shows the feature annotations. Annotations in black are based on comparisons to the Adams or NIST17 libraries. Annotations in blue are based on comparisons to authentic standards, or NMR of the purified compound.

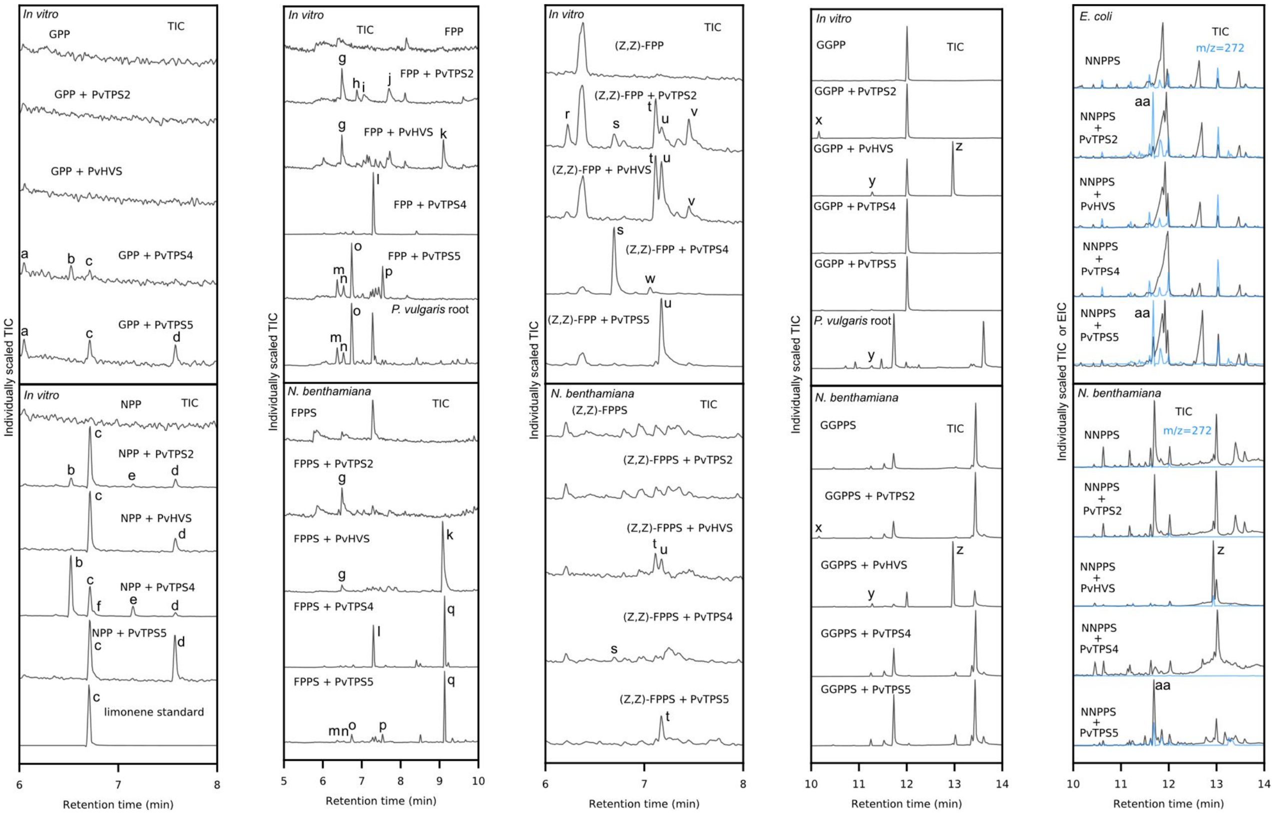

Key

**a**	myrcene	**o**	β-barbatene
**b**	α-terpinene	**p**	unidentified sesquiterpene 2
**c**	limonene	**q**	unidentified sesquiterpene 3
**d**	p-mentha-2,4(8)-diene	**r**	unidentified sesquiterpene 4
**e**	unidentified monoterpene 1	**s**	unidentified sesquiterpene 5
**f**	phellandrene	**t**	β-bisabolene
**g**	(E)-β-farnesene	**u**	(Z)-γ-bisabolene
**h**	(Z,E)-α-farnesene	**v**	unidentified sesquiterpene 6
**i**	(E,E)-α-farnesene	**w**	α-zingiberene
**j**	(E)-nerolidol	**x**	unidentified diterpene 1
**k**	bisabolol	**y**	unidentified diterpene 2
**l**	δ-cadinene	**z**	11-hydroxy vulgarisane
**m**	α-barbatene	**aa**	unidentified diterpene 3
**n**	unidentified sesquiterpene 1		

Fig. S4. Mass spectra of enzyme products and selected reference spectra.

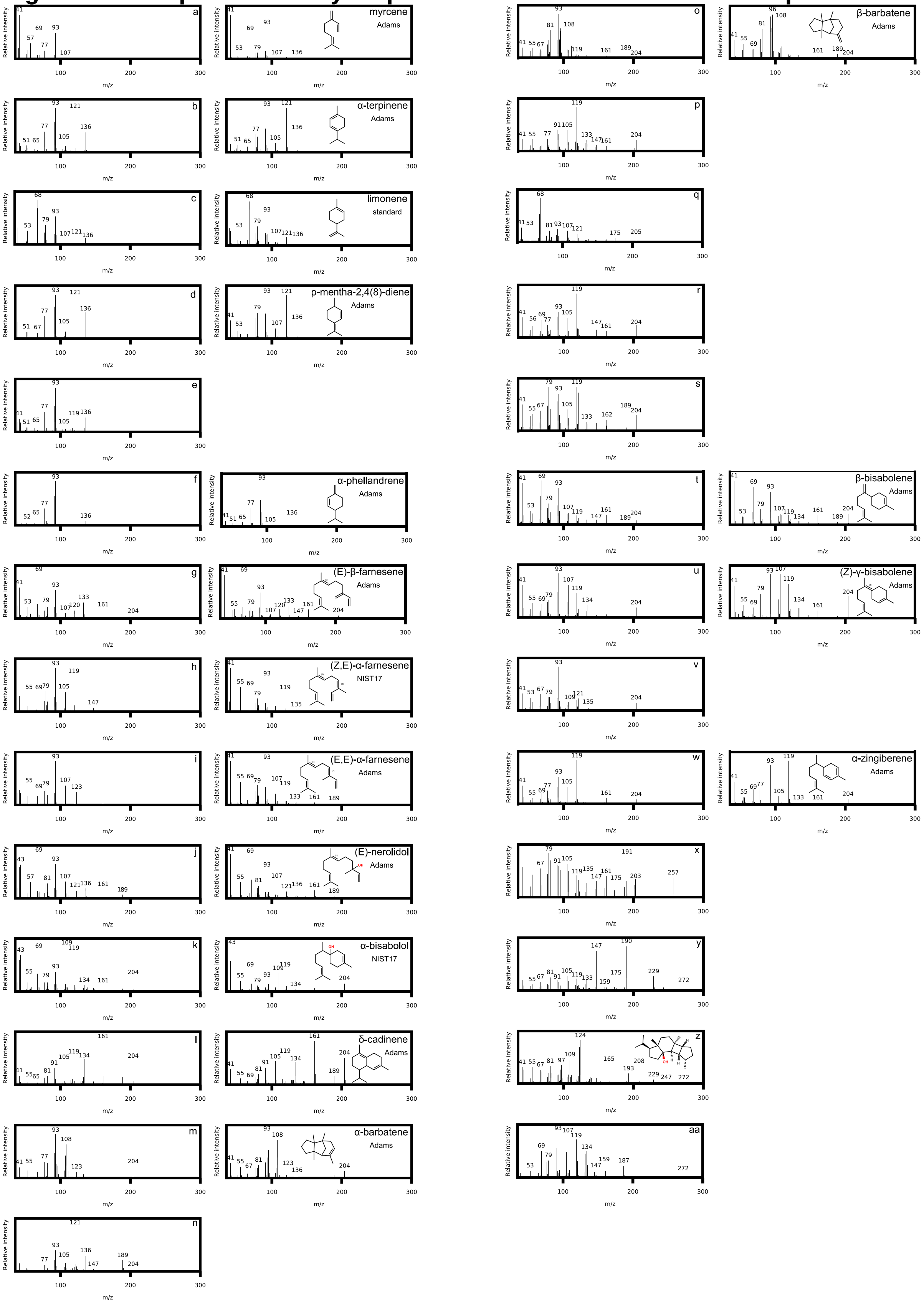

Fig. S5. Peak q only appears when PvTPS4 or PvTPS5 is co-expressed with FPPS in *N. benthaminana*.

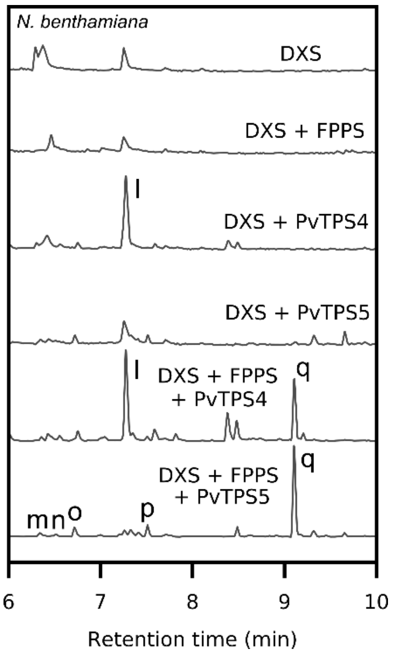

Fig. S6. NMR spectra of PvHVS major product from GGPP, identified as 11-hydroxy vulgarisane.

^{1}H NMR (500 MHz, cdcl$_3$) δ 0.87 (s, 3H, H-16), 0.89 (d, *J* = 6.6 Hz, 3H, H-20), 0.95 (d, *J* = 6.7 Hz, 3H, H-15), 0.96 (d, *J* = 6.5 Hz, 3H, H-19), 1.02 (d, *J* = 1.2 Hz, 1H, OH-11), 1.07 (s, 3H, H-17), 1.17 (ddd, *J* = 13.6, 4.1, 2.8 Hz, 1H, H-8a), 1.30 (m, 1H, H-4a), 1.31 (m, 1H, H-12a), 1.35 (m, 1H, H-13a), 1.44 (m, 2H, H-5a, H-12b), 1.46 (m, H-14), 1.53 (d, *J* = 27.4 Hz, 1H, H-8b), 1.58 (m, 2H, 9a, H-13b), 1.65 (m, 2H, H-5b, H-18), 1.67 (m, 1H, H-4b), 1.70 (d, *J* = 6.4 Hz, 1H, H-1), 1.81 (m, 2H, H-3, H-9b), 2.20 (t, *J* = 8.0 Hz, 1H, H-6), 2.38 (q, *J* = 6.9 Hz, 1H, H-2).

^{13}C NMR (126 MHz, cdcl$_3$) δ 14.33 (C-15), 21.14 (C-17), 22.76 (C-20), 23.51 (C-16), 23.97 (C-19), 27.03 (C-5), 27.33 (C-13), 28.17 (C-18), 32.69 (C-8), 34.02 (C-4), 35.04 (C-9), 35.06 (C-7), 36.67 (C-3), 41.19 (C-2), 42.02 (C-12), 44.18 (C-6), 44.81 (C-10), 46.19 (C-1), 55.88 (C-14), 81.60 (C-11).

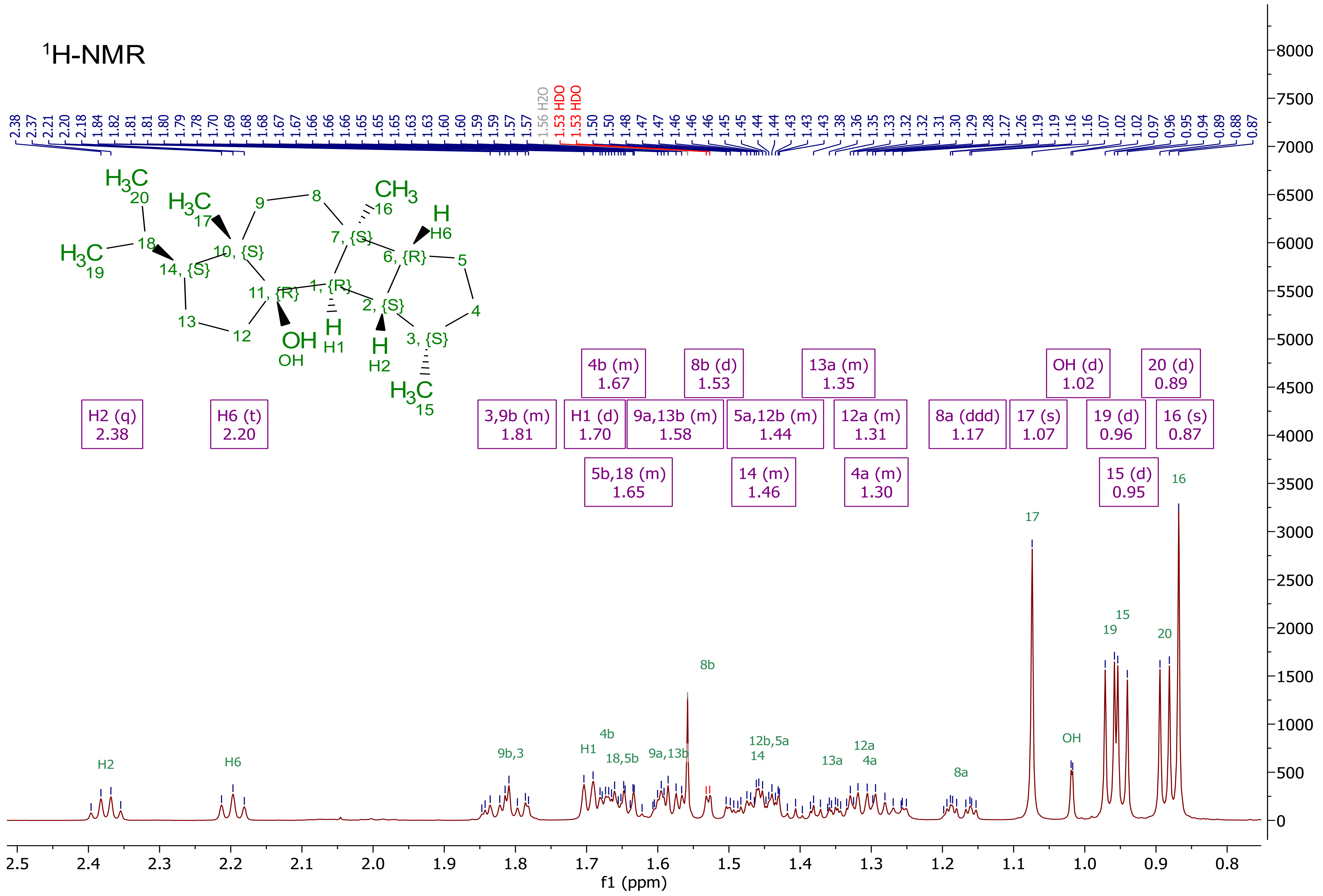

¹H-NMR
2.38 2.37 2.21 2.20 2.18 1.84 1.82 1.81 1.81 1.80 1.79 1.78 1.70 1.69 1.68 1.68 1.67 1.67 1.66 1.66 1.66 1.65 1.65 1.65 1.63 1.63 1.60 1.60 1.59 1.59 1.57 1.57 1.56 H2O 1.53 HDO 1.53 HDO 1.50 1.50 1.48 1.47 1.47 1.46 1.46 1.46 1.45 1.45 1.44 1.44 1.43 1.43 1.43 1.38 1.36 1.35 1.33 1.32 1.31 1.30 1.29 1.28 1.27 1.26 1.19 1.19 1.16 1.16 1.07 1.02 1.02 0.97 0.96 0.95 0.94 0.89 0.88 0.87
H2 (q) 2.38
H6 (t) 2.20
3,9b (m) 1.81
4b (m) 1.67
H1 (d) 1.70
5b,18 (m) 1.65
8b (d) 1.53
9a,13b (m) 1.58
13a (m) 1.35
5a,12b (m) 1.44
14 (m) 1.46
12a (m) 1.31
4a (m) 1.30
8a (ddd) 1.17
17 (s) 1.07
OH (d) 1.02
19 (d) 0.96
15 (d) 0.95
20 (d) 0.89
16 (s) 0.87
f1 (ppm)

^{13}C-NMR

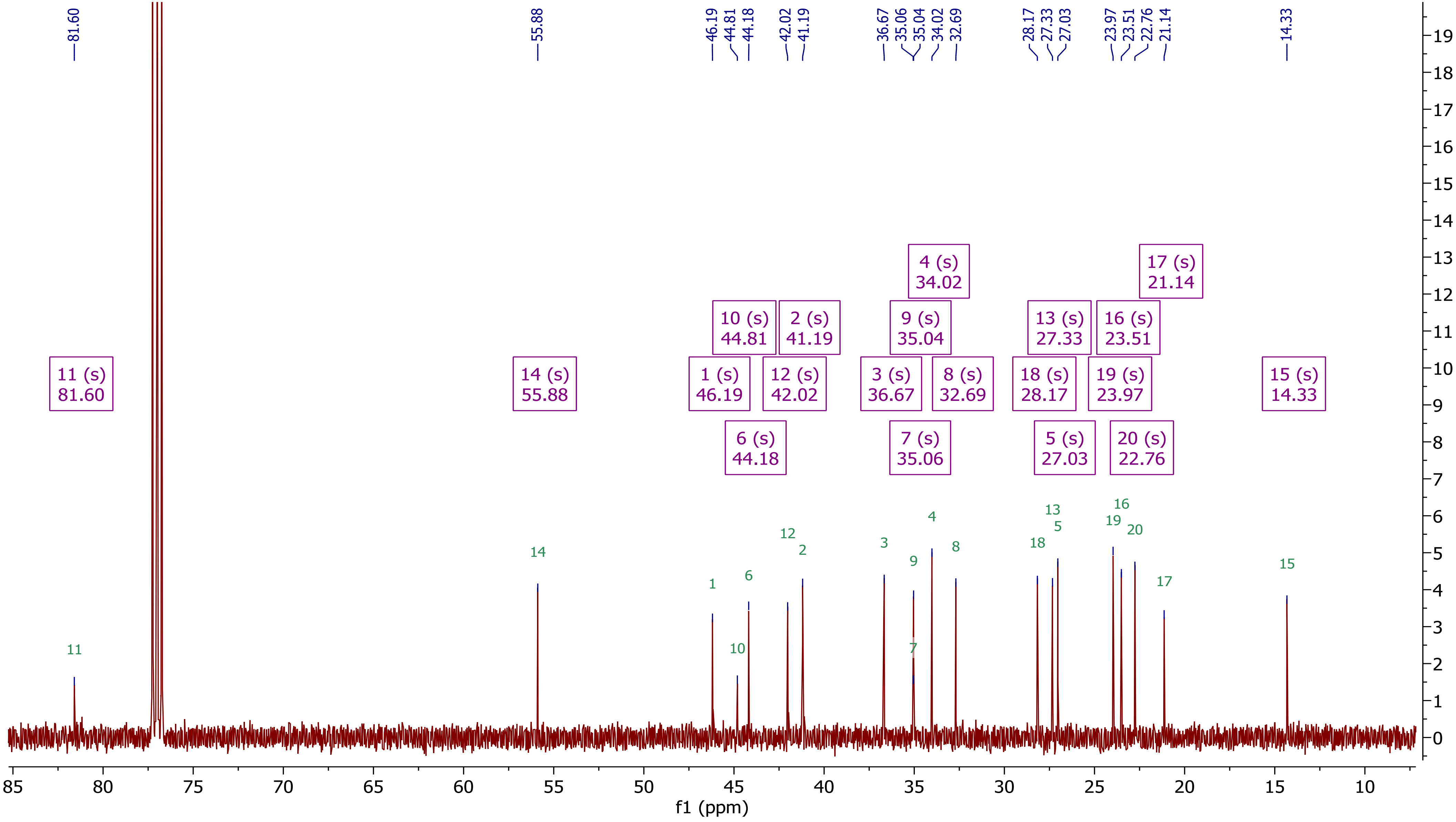

81.60
55.88
46.19
44.81
44.18
42.02
41.19
36.67
35.06
35.04
34.02
32.69
28.17
27.33
27.03
23.97
23.51
22.76
21.14
14.33
4 (s) 34.02
17 (s) 21.14
10 (s) 44.81
2 (s) 41.19
9 (s) 35.04
13 (s) 27.33
16 (s) 23.51
11 (s) 81.60
14 (s) 55.88
1 (s) 46.19
12 (s) 42.02
3 (s) 36.67
8 (s) 32.69
18 (s) 28.17
19 (s) 23.97
15 (s) 14.33
6 (s) 44.18
7 (s) 35.06
5 (s) 27.03
20 (s) 22.76
f1 (ppm)

COSY

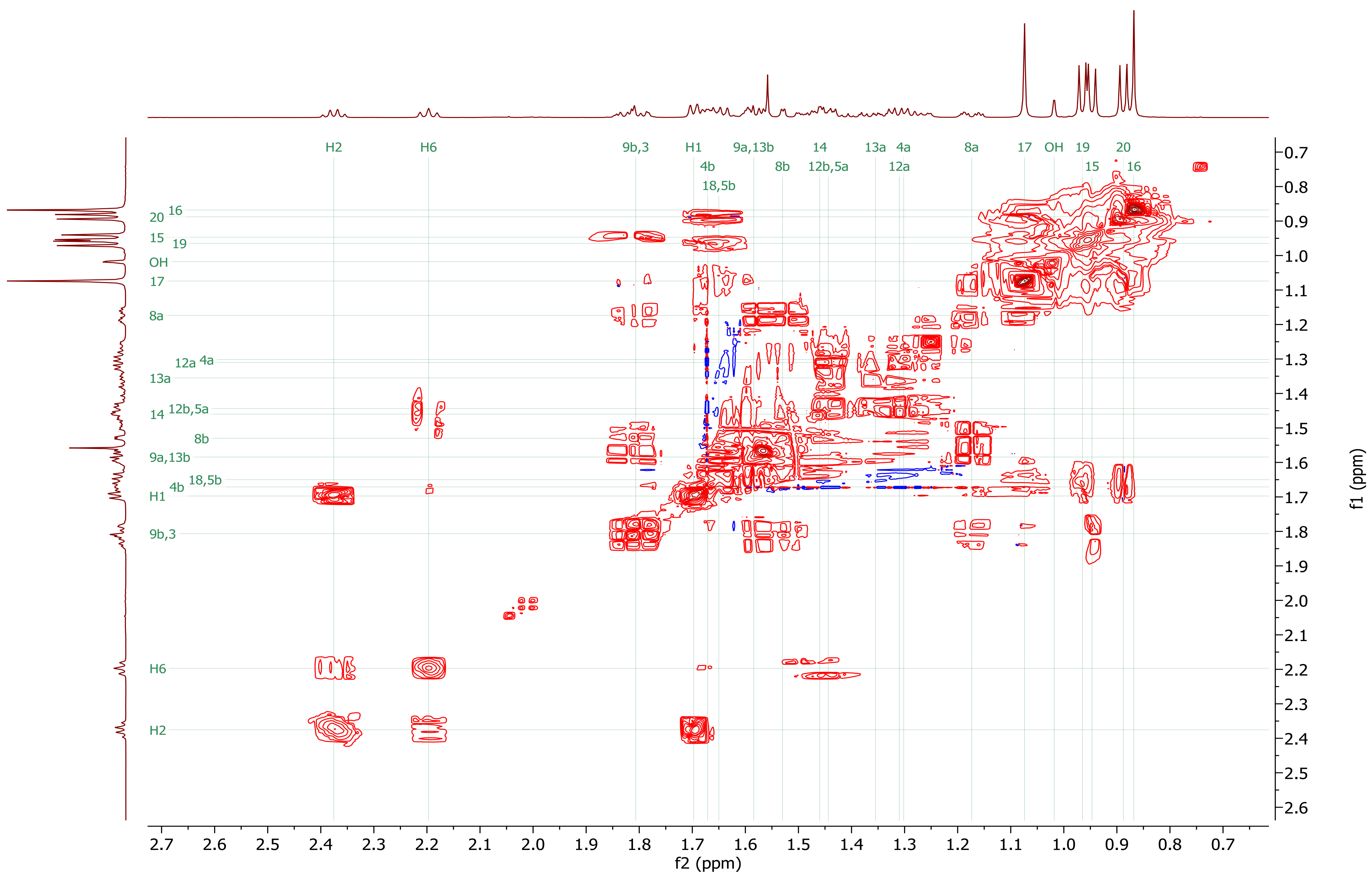

H2
H6
9b,3
H1
4b
18,5b
9a,13b
8b
14
12b,5a
13a
4a
12a
8a
17
OH
19
15
20
16
12a
13a
9a,13b
f2 (ppm)
f1 (ppm)

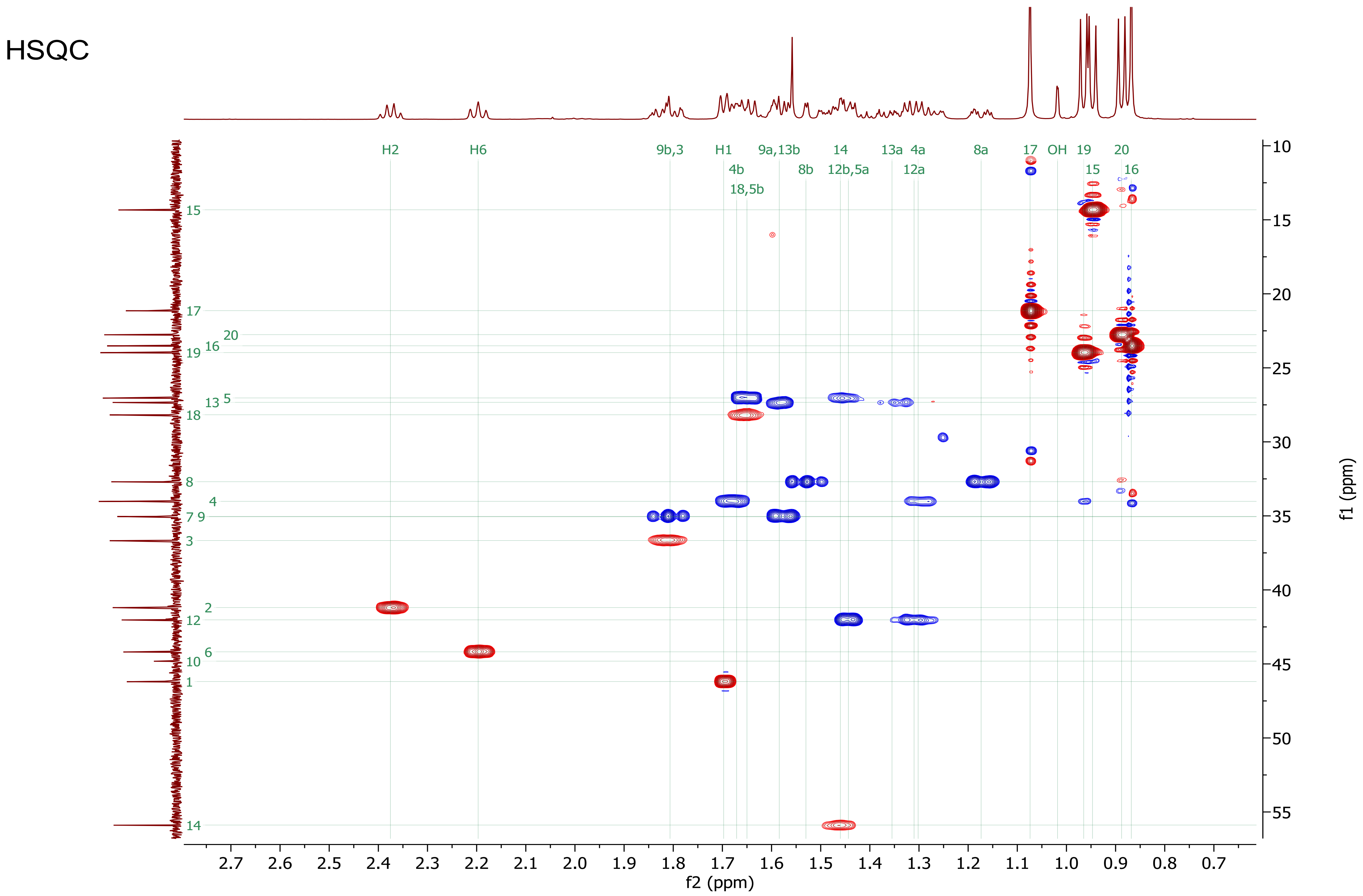
HSQC
H2
H6
9b,3
H1
4b
18,5b
9a,13b
8b
14
12b,5a
13a
4a
12a
8a
17
OH
19
15
20
16
15
17
20
16
19
13
5
18
8
4
7 9
3
2
12
6
10
1
14
f2 (ppm)
f1 (ppm)

HMBC

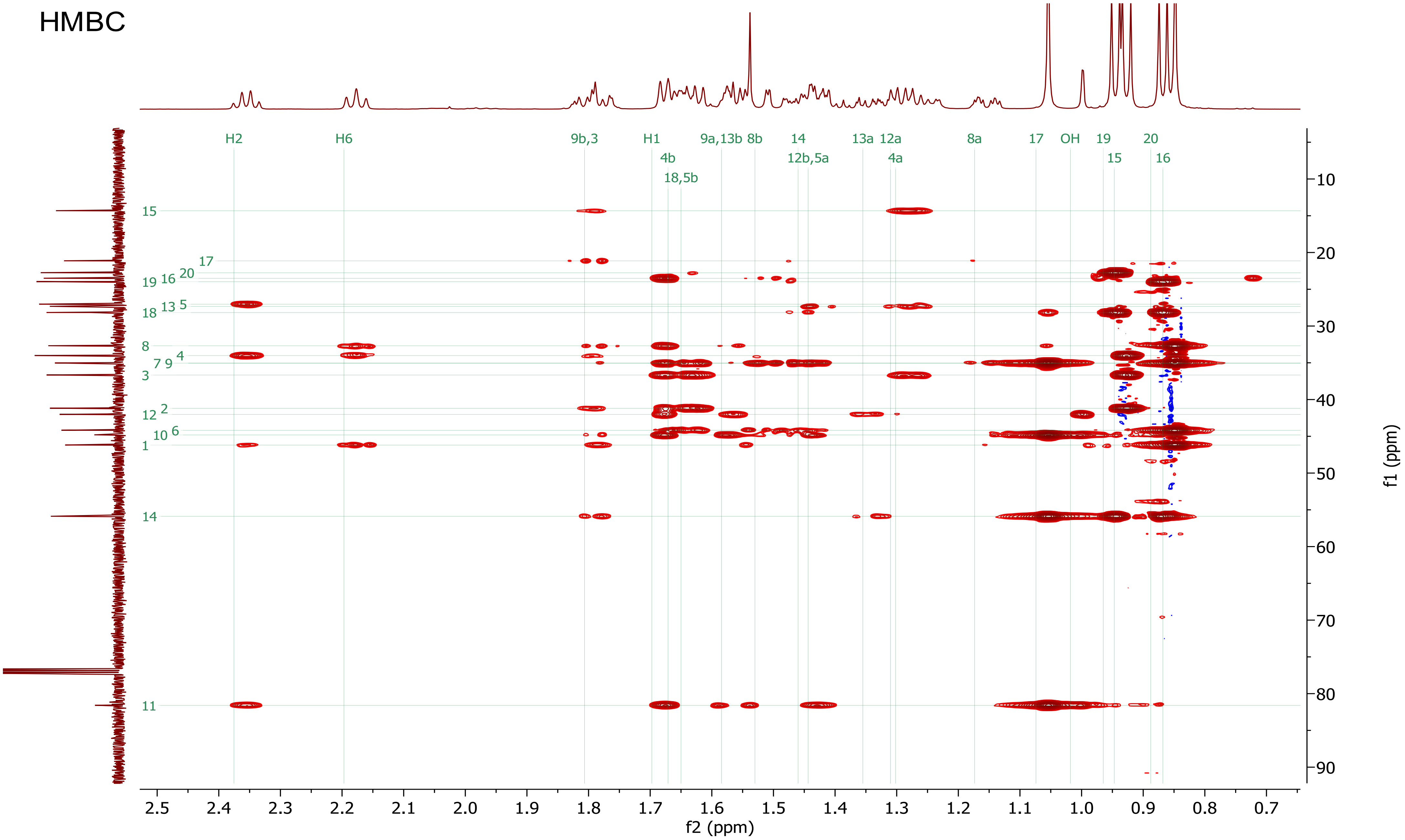
H2
H6
9b,3
H1
4b
18,5b
9a,13b
8b
14
12b,5a
13a
12a
4a
8a
17
OH
19
15
20
16
15
17
19 16 20
18 13 5
8
7 9 4
3
12 2
10 6
1
14
11
f2 (ppm)
f1 (ppm)

NOESY

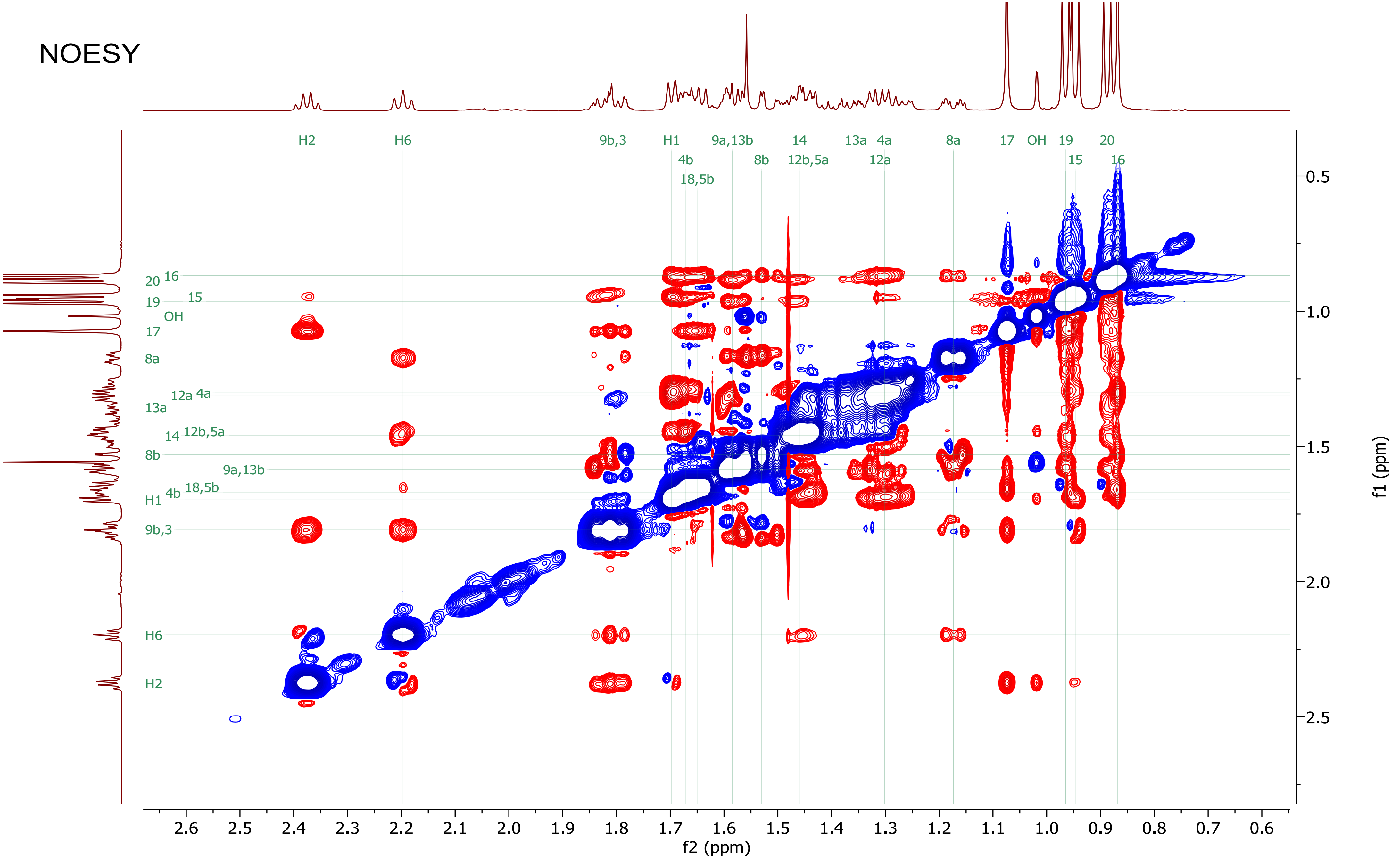

H2
H6
9b,3
H1
4b
18,5b
9a,13b
8b
14
12b,5a
13a
4a
12a
8a
17
OH
19
15
20
16
2.6 2.5 2.4 2.3 2.2 2.1 2.0 1.9 1.8 1.7 1.6 1.5 1.4 1.3 1.2 1.1 1.0 0.9 0.8 0.7 0.6
f2 (ppm)
0.5 1.0 1.5 2.0 2.5
f1 (ppm)

Fig. S7. 11-Hydroxyvulgarisane (z) in *P. vulgaris*. A 70% methanol extract of root tissue was subjected to non-targeted UHPLC/MS^E analysis using a 31-min chromatographic separation. (**a**) Extracted ion chromatogram for *m/z* 273.3 ([M+H-H_2O]$^+$) for the crude *P. vulgaris* extract and (**b**) extracted ion chromatogram for m/z 273.3 for the purified 11-hydroxy vulgarisane, (**c**) MS/MS spectrum of the purified 11-hydroxyvulgarisane. The identity of purified 11-hydroxyvulgarisane was confirmed by NMR.

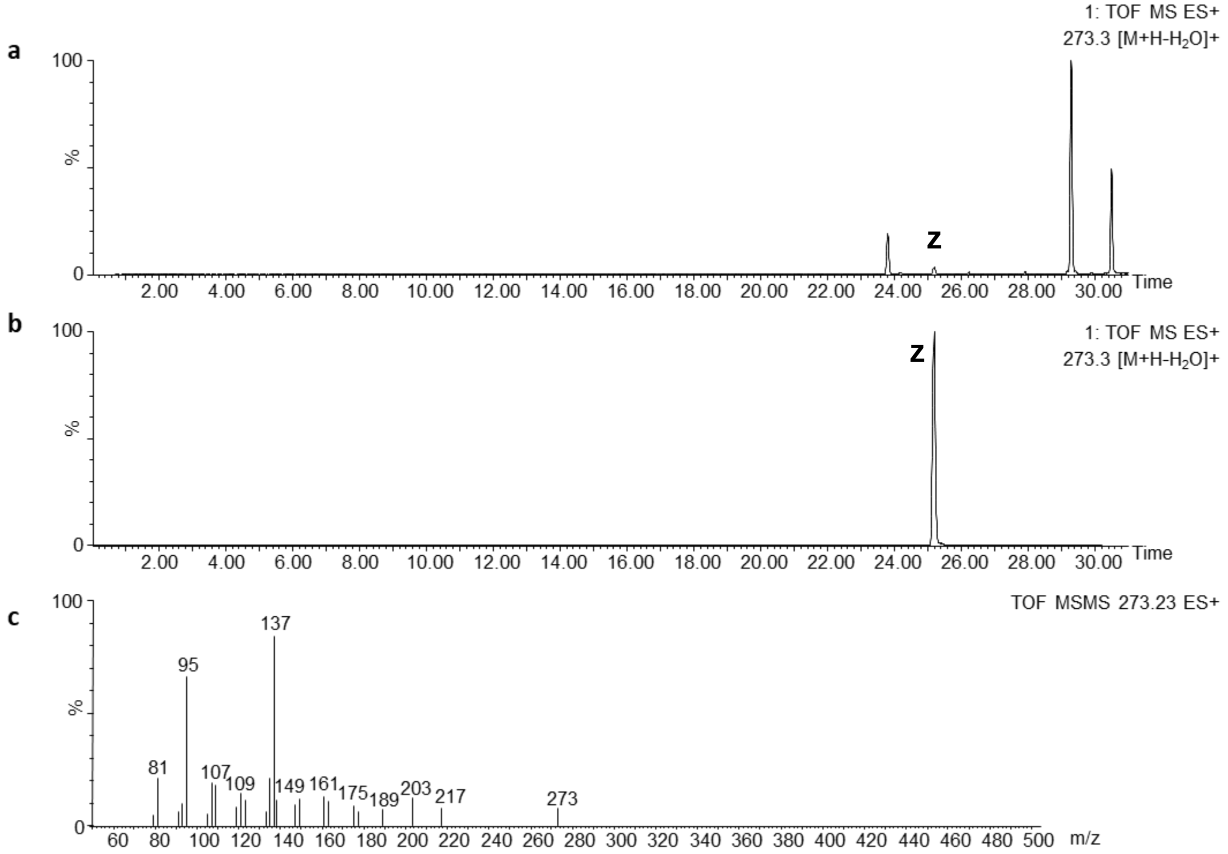

Table S2. Exact masses and relative abundance for ions of vulgarisin-related compounds detected by UHPLC/MS^E in *P. vulgaris* root extracts.

**Annotated Metabolite**	**Formula**	**Retention time [min]**	**Calculated m/z**	**Experimental m/z**	**Precursor and fragment ion(s) m/z and relative ion abundances (%)**
11-Hydroxy vulgarisane (**Fig. S7 c**)	$C_{20}H_{34}O$	25.19	273.2582 $[M+H-H_2O]^+$	273.2585 $[M+H-H_2O]^+$	273.26 (10), 217.20 (10), 203.18 (15), 189.16 (9), 137.13 (100), 107.08 (24), 95.08 (85)
Vulgarisin A isomer 1 (**Fig. S8 b, top**)	$C_{28}H_{46}O_6$	17.17	501.3192 $[M+Na]^+$	501.3191 $[M+Na]^+$	501.32 (15), 461.33 (100), 373.27 (51), 285.22 (74), 267.21 (21)
Vulgarisin A isomer 2 (**Fig. S8 b, bottom**)	$C_{28}H_{46}O_6$	19.88	501.3192 $[M+Na]^+$	501.3184 $[M+Na]^+$	501.32 (34), 461.33 (100), 373.27 (33), 285.22 (58), 267.21 (20)

Fig. S8. Vulgarisin A and D isomers in *P. vulgaris*. A 70% methanol extract of root tissue was subjected to non-targeted UHPLC/MS^E analysis using a 31-min chromatographic separation and electrospray ionization in positive-ion mode. (**a**) Extracted ion chromatogram for *m/z* 501.3 ([M+Na]$^+$) of the *P. vulgaris* extract is shown in function 1 (**b**). The mass spectra of the two peaks are shown, displaying prominent [M+H-H_2O]$^+$ ions in each case at *m/z* 461 and subsequent loss of isobutyric acid (88 Da) at *m/z* 373. Note that the mass spectra of the two isomers are consistent with the previously reported spectra for vulgarisin A (Lou *et al.*, 2014) and vulgarisin D (Lou *et al.*, 2017).

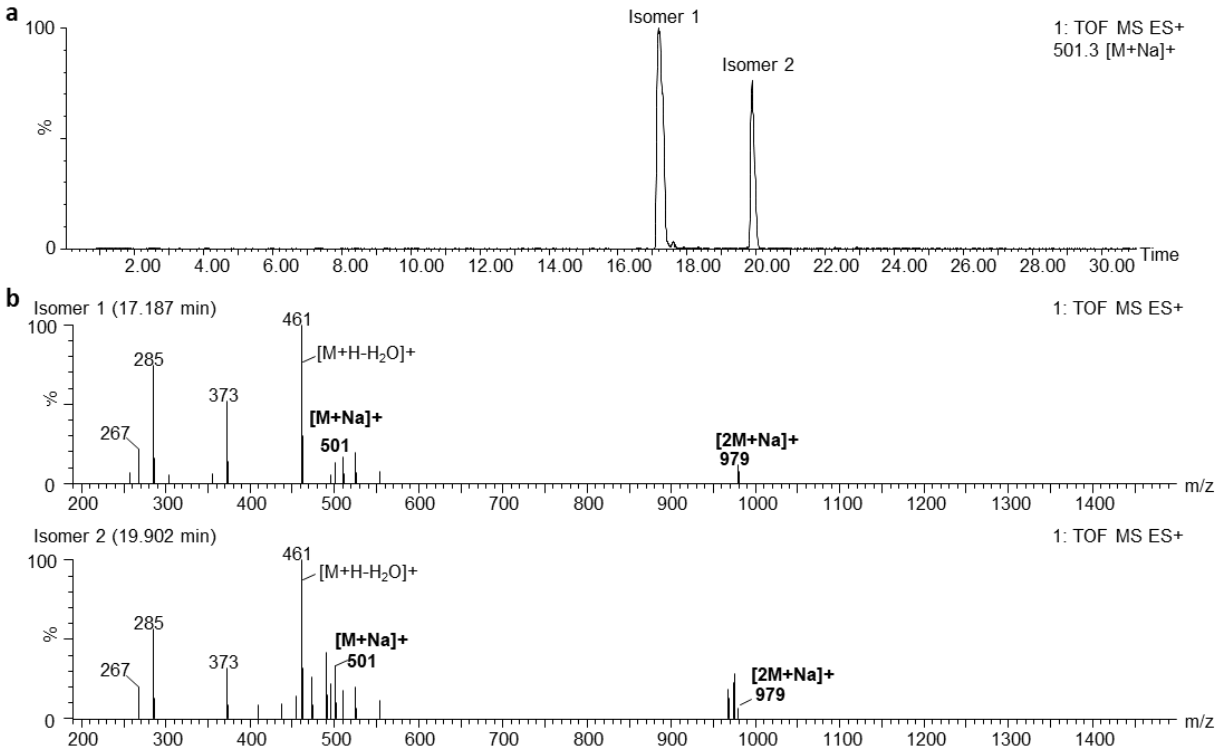

Additional references:

Fu S, Ma Y, Yao H, Xu Z, Chen S, Song J, Au KF, Berger B. **2018**. IDP-denovo: de novo transcriptome assembly and isoform annotation by hybrid sequencing. *Bioinformatics* **34**: 2168–2176.

Hamberger B, Ohnishi T, Hamberger B, Séguin A, Bohlmann J. **2011**. Evolution of diterpene metabolism: Sitka spruce CYP720B4 catalyzes multiple oxidations in resin acid biosynthesis of conifer defense against insects. *Plant Physiology* **157**: 1677–1695.

Hoang NV, Furtado A, Mason PJ, Marquardt A, Kasirajan L, Thirugnanasambandam PP, Botha FC, Henry RJ. **2017**. A survey of the complex transcriptome from the highly polyploid sugarcane genome using full-length isoform sequencing and de novo assembly from short read sequencing. *BMC Genomics* **18**.

Lou H-Y, Jin L, Huang T, Wang D-P, Liang G-Y, Pan W-D. **2017**. Vulgarisins B–D, three novel diterpenoids with a rare skeleton isolated from *Prunella vulgaris* Linn. *Tetrahedron Letters* **58**: 401–404.

Lou H, Zheng S, Li T, Zhang J, Fei Y, Hao X, Liang G, Pan W. **2014**. Vulgarisin A, a new diterpenoid with a rare 5/6/4/5 ring skeleton from the Chinese medicinal plant *Prunella vulgaris*. *Organic Letters* **16**: 2696–2699.

Salmela L, Rivals E. **2014**. LoRDEC: accurate and efficient long read error correction. *Bioinformatics* **30**: 3506–3514.
